# Delayed Diagnosis of Transthyretin Cardiac Amyloidosis Is Associated With Heart Failure Hospitalizations and Mortality

**DOI:** 10.1016/j.jacadv.2026.103019

**Published:** 2026-07-22

**Authors:** Gabriela Spencer-Bonilla, Jun Fan, Anubodh Sunny Varshney, Paul Cheng, Natasha Din, Fatima Rodriguez, Marie Davies, Mia A. Papas, Joanna Huang, John Venditto, Ronald M. Witteles, Paul Heidenreich, Kevin Alexander, Alexander T. Sandhu

**Affiliations:** a Stanford Cardiovascular Institute, Stanford University School of Medicine, Stanford, California, USA; b Veteran’s Affairs Palo Alto Healthcare System, Palo Alto, California, USA; c U.S. Medical Affairs, BioPharmaceutical Medical, AstraZeneca, Wilmington, Delaware, USA; d Division of Cardiology, Department of Medicine, University of California Los Angeles, Los Angeles, California, USA; e Greater Los Angeles VA Healthcare System, Los Angeles, California, USA

**Keywords:** amyloidosis, cardiomyopathy, diagnostic delay, transthyretin

## Abstract

**Background:**

Transthyretin cardiac amyloidosis (ATTR-CM) is a progressive, underdiagnosed cause of heart failure (HF). Diagnostic delays may increase cardiac injury at treatment initiation, but the relationship between delay and outcomes remains poorly defined.

**Objectives:**

The purpose of this study was to evaluate time from HF diagnosis to ATTR-CM diagnosis as a proxy for disease progression and its association with HF hospitalization (HFH) and mortality.

**Methods:**

This retrospective cohort study used Medicare fee-for-service and Veterans Health Administration (VHA) data. We identified patients diagnosed with ATTR-CM between 2016 and 2022 using a validated algorithm based on diagnoses and medications. Time-to-diagnosis was defined as days between each patient's first HF diagnosis and first amyloid diagnosis. Using multivariable Cox models, we evaluated its association with death or HFH.

**Results:**

We identified 7,770 Medicare beneficiaries and 2,557 Veterans with HF and ATTR-CM. Median age at diagnosis was 81 years in both cohorts (Medicare IQR: 76-86; VHA IQR: 74-87); women comprised 1,775 (22.8%) of Medicare and 13 (0.5%) of VHA patients. Median time-to-diagnosis was 494 days (IQR: 63-1,340) for Medicare and 490 days (IQR: 69-1,286) for VHA. After adjustment for sociodemographics, each 1-year delay was associated with a 7% increased risk of the primary outcome (Medicare HR: 1.07; 95% CI: 1.06-1.08; VHA HR: 1.07; 95% CI: 1.05-1.09). Results were similar after adjusting for comorbidities.

**Conclusions:**

Across 2 real-world populations, diagnostic delay in ATTR-CM is a clinically meaningful marker of disease progression, with longer delays associated with increased HFH and mortality.

Transthyretin cardiac amyloidosis (ATTR-CM) is a fatal yet often underdiagnosed form of cardiomyopathy that results from the deposition of misfolded transthyretin (TTR) protein in the heart tissue, leading to cardiac dysfunction.^1^, ^2^, ^3^, ^4^ The clinical course in ATTR-CM is typically progressive as ongoing amyloid deposition leads to worsening cardiac function and decreased functional status over time. Given its progressive nature, early detection of ATTR-CM is critical to reduce disease morbidity. However, ATTR-CM symptoms may be attributed to more prevalent cardiac and systemic conditions, leading to misdiagnosis or delayed recognition of ATTR-CM.^1^, ^2^, ^3^^,^^5^ Therefore, diagnostic delay in ATTR-CM may reflect a prolonged period of untreated disease progression, during which patients accumulate cardiac damage. Without timely diagnosis, patients may undergo ineffective treatments, while the underlying cause of their condition remains unaddressed and progresses.^6^^,^^7^ Thus, time to diagnosis may serve not only as a health care delivery metric but also as a clinically meaningful marker of prognosis at presentation.^2^

We previously described the time from heart failure (HF) to ATTR-CM diagnosis and the predictors of delayed diagnosis of ATTR-CM among Medicare beneficiaries and Veterans receiving care in the Veterans Health Administration (VHA). In the present study, we examine the relationship between diagnostic delay as a marker of disease progression and subsequent clinical outcomes.

## Methods

### Data

For Medicare fee-for-service beneficiaries, we used administrative claims data from 2008 to 2022. For VHA patients, we used VHA electronic health record data and linked data from Medicare claims and community care data between 2008 and 2022. The data include demographics, outpatient and inpatient encounters, diagnoses, procedures, and pharmacy dispensing records. HF hospitalizations (HFHs) were identified via International Classification of Diseases (ICD) codes. For Medicare beneficiaries, mortality data were extracted from Medicare enrollment files. We identified Veteran deaths via the Mortality Data Repository. Among Veterans, we identified cardiovascular death via cause of death data from death certificates, which are available through the VHA Mortality Data Repository but were not available in our Medicare research files.^8^ This study was approved by the Institutional Review Board of Stanford University.

### Algorithm for ATTR-CM

We previously developed and described an algorithm to identify ATTR-CM using structured diagnosis and medication data in the VHA database.^5^ The algorithm identified individuals with HF and amyloid diagnoses without other non-ATTR causes for their amyloid. Supplemental Table 1 details the diagnosis codes and medications used in the algorithm. The algorithm had a sensitivity and positive predictive value of 84% and 94%, respectively.

### Study population

We first identified patients with an HF diagnosis in addition to an amyloidosis diagnosis between 2016 and 2022. The time period of 2016 to 2022 was selected in order to have adequate follow-up while also being an analysis of contemporary practice patterns. We identified those with HF based on one inpatient principal diagnosis or 2 total HF diagnoses within a 1-year time window.^9^ We then identified patients with incident ATTR-CM between 2016 and 2022 using the algorithm described above.^5^ We excluded patients in whom the incident HF diagnosis was more than 10 years before the incident amyloid diagnosis. This exclusion was based on the assumption that the cardiomyopathy may be secondary to an alternate etiology given the high mortality of untreated cardiac amyloidosis.^10^ We included patients in whom the amyloidosis diagnosis preceded the HF diagnosis but was within 1 year given this suggested timely diagnosis of ATTR-CM.

Among the Medicare cohort, we required at least 3 years of continuous enrollment in Medicare Part A, B, and C prior to their first ATTR-CM and HF diagnoses to ensure we were capturing incident amyloid. We also required 6 months of continuous enrollment in Medicare Part D prior to their ATTR-CM diagnosis given medication therapy was included in the ATTR-CM identification algorithm. We excluded any Medicare beneficiaries with evidence of VHA health care payment to remove duplicates across both cohorts. In the VHA cohort, to identify active VHA users and minimize missing data, we excluded Veterans without any outpatient visits or medication prescription in the VHA in the year prior to incident ATTR-CM diagnosis.

### Outcomes

The primary outcome of interest was the time to the composite outcome of HFH and all-cause death. Secondary outcomes included the time to HFH and the time to all-cause death as individual outcomes. Among the VHA cohort, we also evaluated the time to cardiovascular death as a secondary outcome.

### Study variables

The primary exposure was the time to diagnosis for each patient using the number of days between their first HF diagnosis and their incident amyloidosis diagnosis. A secondary exposure was the number of days between first loop diuretic prescription and incident amyloidosis diagnosis.

Additional patient-level variables included sociodemographic and clinical characteristics. Sociodemographic variables included age, sex, race, ethnicity, marital status, CDC Social Vulnerability Index (based on zip code), rural/urban classification, drive time to primary care, and drive time to specialty care.

We identified medical comorbidities and prior procedures using a 3-year look back prior to the incident amyloidosis diagnosis. We identified medical comorbidities based on ICD-9 and ICD-10 revision diagnostic codes (see Supplemental Table 2). Comorbidities collected included those that could represent an alternate cardiomyopathy etiology (alcohol use disorder, coronary artery disease, drug use disorder), those associated with ATTR amyloidosis (aortic stenosis, atrial fibrillation, atrial arrhythmias, carpal tunnel, complete heart block, peripheral neuropathy, or spinal stenosis), and other general comorbidities (chronic kidney disease, chronic obstructive pulmonary disease, depression, diabetes mellitus, hypertension, liver disease, psychotic disorder, thyroid disease, Charlson Comorbidity Index, and frailty).

We identified prior medical procedures using Current Procedural Terminology codes (Supplemental Table 3). We identified primary care, cardiology, and neurology visits based on Health Care Financing Administration codes. Medication fills within 6 months prior to the incident amyloid and incident HF dates were extracted from Medicare Part D prescription fills for the Medicare cohort. For the VHA, we captured medications filled in the VHA, other medications entered in the VHA electronic health record, and prescriptions filled by Medicare Part D. We identified the date of first outpatient loop diuretic prescription for each patient since 2008. We also identified ATTR-CM-directed medical therapies (tafamidis, patisiran, or inotersen) within 6 months after ATTR-CM diagnosis.

We identified HFHs based on acute care hospitalizations with a principal diagnosis of HF. For Medicare patients, HF hospitalizations were identified via MEDPAR files. We restricted to acute-care, short-stay hospitals. For the VHA cohort, we also included VHA and community care hospitalizations.

### Statistical analyses

We described each cohort’s characteristics via median and IQR for continuous, non-normally distributed variables, mean and SD for continuous, normally distributed variables, and frequency and percentages for categorical values. We stratified patient characteristics in each cohort based on a binary measure of diagnostic delay; we defined ≥6 months as a substantial delay given prior data regarding the substantial morbidity of ATTR-CM. We used 2-sample *t*-test for normally distributed variables, the Wilcoxon rank sum test for non-normal variables, and chi-square for categorical variables.

We performed a series of multivariable Cox regression models to evaluate the association between time to diagnosis as a continuous measure and the outcomes of interest. We used a continuous measure with assumed linearity for interpretability. We also modeled the time to diagnosis as a restricted cubic spline with 4 knots and as a categorical measure: time to diagnosis <6 months, ≥6 and < 2 years, and ≥2 years. We used 6 months as a cutoff for timely diagnosis to account for a reasonable duration between initial HF diagnosis and referral to a specialist for consideration of ATTR-CM testing.^3^^,^^11^ To prevent immortal time bias, the index date (time zero) was the date of incident amyloidosis diagnosis. The primary analysis was adjusted for sociodemographics (age, sex, neighborhood social vulnerability, and rurality) but not comorbidities because the time to diagnosis may impact the severity of comorbidities. For example, progressive amyloid may lead to chronic kidney disease. We performed the following nested models: 1) unadjusted; 2) adjusted for sociodemographics: age, sex, rurality, and CDC Social Vulnerability Index;^12^ and 3) adjusted for sociodemographics, the Charlson Comorbidity Index, and comorbidities. We modeled continuous characteristics as restricted cubic splines with 4 knots. We repeated the survival models for each secondary outcome. For the HFH outcome, we censored at the time of death. For Medicare beneficiaries, we also censored outcomes at the time of Medicare Part A enrollment gaps. The Cox models violated the global test for proportional hazards; therefore, our HRs represent the average ratio over the period of follow-up.^13^

The analysis was repeated with multiple secondary analyses. First, we repeated the analysis using the time from first loop diuretic prescription to ATTR-CM diagnosis as the exposure of interest. Second, we repeated the analysis with an interaction between the time to diagnosis and the time period of ATTR-CM diagnosis, stratified by the year before vs after the Food and Drug Administration approval of ATTR-CM therapies (2019). Third, we repeated the analysis after excluding individuals with ATTR-CM diagnosis preceding HF diagnosis.

We used Stata 16 (StataCorp LLC) with a threshold of P £0.05 for statistical significance. Data access for this project is not available upon request. This study was approved by the Stanford University Institutional Review Board and follows the STROBE (Strengthening the Reporting of Observational Studies in Epidemiology) reporting guidelines.

## Results

### ATTR-CM cohorts

The details of the cohorts have been previously described.^14^^,^^15^ The final cohorts comprised 7,770 Medicare enrollees (Supplemental Figure 1) and 2,557 Veterans (Supplemental Figure 2) with ATTR-CM.

### Medicare cohort

The median age was 81 years, with 23% (1,775) of enrollees being women. Most enrollees were non-Hispanic White (5,789; 75%) or Black (1,522; 20%). The most prevalent comorbidities at the time of amyloidosis diagnosis were hypertension (6,597; 85%), coronary artery disease (5,225; 67%), and atrial fibrillation or flutter (4,891; 63%). The most prescribed cardiac medications were loop diuretics (4,696; 60%), beta-blockers (4,600; 59%), renin-angiotensin system inhibitors (3,871; 50%), and anticoagulants (3,859; 50%). Baseline characteristics at the time of amyloidosis diagnosis are provided in Table 1.

**Table 1:** Characteristics at the Time of Incident Amyloid Diagnosis

	Total Medicare Cohort (N = 7,770)	Shorter Time to Diagnosis (n = 2,778)	Longer Time to Diagnosis (n = 4,992)	Total VHA Cohort (n = 2,557)	Shorter Time to Diagnosis (n = 902)	Longer Time to Diagnosis (n = 1,655)
Sociodemographic characteristics						
Age, median (IQR) in y	81 (76,86)	80 (75,85)	82 (77,86)	80.5 (74-87)	80 (73-87)	81.1 (74-88)
Sex, (%)						
Female	1,775 (22.8%)	602 (21.7%)	1,173 (23.5%)	13 (0.5%)	7 (0.8%)	6 (0.4%)
Male	5,995 (77.2%)	2,176 (78.3%)	3,819 (76.5%)	2,544 (99.5%)	895 (99.2%)	1,649 (99.6%)
Race and ethnicity, n (%)						
Black or African American	1,522 (19.6%)	517 (18.6%)	1,005 (20.1%)	764 (29.9%)	282 (31.3%)	482 (29.1%)
Hispanic/Latino/a	227 (2.9%)	75 (2.7%)	152 (3.0%)	124 (4.8%)	45 (5.0%)	79 (4.8%)
Non-Hispanic White	5,789 (74.5%)	2077 (74.8%)	3,712 (74.4%)	1,440 (56.3%)	488 (54.1%)	952 (57.5%)
Other^a^	162 (2.1%)	69 (2.5%)	93 (1.9%)	37 (1.4%)	9 (1.0%)	28 (1.7%)
Missing	192 (7.5%)	40 (1.4%)	30 (0.6%)	192 (7.5%)	78 (8.6%)	114 (6.9%)
Regional designation, n (%)^b^						
Non-metropolitan	1,629 (21.0%)	601 (21.6%)	1,028 (20.6)	621 (24.3%)	217 (24.1%)	404 (24.4%)
CDC SVI by quartile^c^						
0.00-0.25	2,049 (26.4%)	742 (26.7%)	1,307 (26.2%)	562 (22.0%)	212 (23.5%)	350 (21.1%)
0.26-0.50	1,975 (25.4%)	714 (25.7%)	1,261 (25.3%)	754 (29.5%)	266 (29.5%)	488 (29.5%)
0.51-0.75	1,933 (25.9%)	702 (25.3%)	1,231 (24.7%)	681 (26.6%)	240 (26.6%)	441 (26.6%)
0.76-1.00	1,786 (23.0%)	620 (22.3%)	1,193 (23.9%)	471 (18.4%)	153 (17.0%)	318 (19.2%)
Missing	27 (0.35%)	742 (26.7%)	1,307 (26.2%)	89 (3.5%)	31 (3.4%)	58 (3.5%)
Medical history						
Comorbidities, n (%)						
Alcohol use disorder	220 (2.8%)	69 (2.5%)	151 (3.0%)	192 (7.5%)	53 (5.9%)	139 (8.4%)
Aortic stenosis	1,759 (22.6%)	425 (15.3%)	1,334 (26.7%)	558 (27.8%)	167 (18.5%)	391 (23.6%)
Atrial fibrillation/flutter	4,891 (63.0%)	1,367 (49.2%)	3,637 (72.9%)	1,578 (61.7%)	424 (47.0%)	1,154 (69.7%)
Carpal tunnel syndrome	1,161 (14.9%)	464 (16.7%)	697 (14.0%)	478 (18.7%)	181 (20.1%)	297 (17.9%)
Chronic kidney disease	3,600 (46.3%)	856 (30.8%)	2,744 (55.0%)	1,314 (51.4%)	336 (37.3%)	978 (59.1%)
Chronic obstructive pulmonary disease	2,305 (29.7%)	551 (19.8%)	1,754 (35.1%)	953 (37.3%)	250 (27.7%)	703 (42.5%)
Complete heart block	539 (6.9%)	93 (3.4%)	446 (8.9%)	183 (7.2%)	34 (3.8%)	149 (9.0%)
Coronary artery disease	5,225 (67.3%)	1,452 (52.3%)	3,773 (75.6%)	1,637 (64.0%)	456 (50.6%)	1,181 (71.4%)
Depression	1,430 (18.4%)	412 (14.8%)	1,018 (20.4%)	694 (27.1%)	215 (23.8%)	479 (28.9%)
Diabetes mellitus	2,180 (28.1%)	614 (22.1%)	1,566 (31.4%)	1,166 (45.6%)	344 (38.1%)	822 (49.7%)
Hypertension	6,597 (84.9%)	2,164 (77.9%)	4,433 (88.8%)	2,405 (94.1%)	805 (89.2%)	1,600 (96.7%)
Liver disease	395 (5.1%)	136 (4.9%)	259 (5.2%)	361 (14.1%)	88 (9.8%)	273 (16.5%)
Peripheral neuropathy	209 (2.69%)	40 (1.4%)	169 (3.4%)	178 (7.0%)	53 (5.9%)	125 (7.6%)
Psychotic disorders/schizophrenia	347 (4.5%)	97 (3.5%)	250 (5.0%)	188 (7.4%)	62 (6.9%)	126 (7.6%)
Spinal stenosis	1,727 (22.2%)	570 (20.5%)	1,157 (23.2%)	513 (20.1%)	179 (19.8%)	334 (20.2%)
Substance use disorder	42 (1.5%)	42 (1.5%)	98 (2.0%)	103 (4.0%)	34 (3.8%)	69 (4.2%)
Thyroid disease	2,053 (26.4%)	616 (22.2%)	1,437 (28.8%)	549 (21.5%)	175 (19.4%)	374 (22.6%)
Frailty	1835 (23.6%)	448 (16.1%)	1,387 (27.8%)	881 (34.5%)	216 (23.9%)	665 (40.2%)
Charlson Comorbidity Index, median (IQR)	5 (3,7)	5 (3,7)	6 (4,8)	5 (3,7)	4 (2,6)	6 (4,7)
Cardiac procedures						
Aortic valve replacement	201 (2.6%)	31 (1.1%)	170 (3.4%)	60 (2.3%)	12 (1.3%)	48 (2.9%)
Atrial ablation	427 (5.5%)	90 (3.2%)	337 (6.8%)	90 (3.5%)	18 (2.0%)	72 (4.4%)
Cardioversion	1,255 (16.2%)	300 (10.8%)	955 (19.1%)	289 (11.3%)	58 (6.4%)	231 (14.0%)
Pacemaker or defibrillator implantation	252 (3.2%)	32 (1.2%)	220 (4.4%)	97 (3.8%)	12 (1.3%)	85 (5.1%)
Medication history, n (%)^d^						
Anticoagulants	3,859 (49.7%)	1,035 (37.3%)	2,824 (54.7%)	1,224 (47.9%)	318 (35.3%)	906 (54.7%)
Antiplatelet medications	776 (10%)	229 (8.2%)	547 (11.0%)	1,414 (55.3%)	487 (54.0%)	927 (56.0%)
Heart failure medications						
ACEi/ARB/ARNi^e^	3,871 (49.8%)	1,404 (50.5%)	2,467 (49.4%)	1,526 (59.7%)	529 (58.6%)	997 (60.2%)
Beta-blockers^f^	4,600 (59.2%)	1,377 (49.6%)	3,223 (64.6%)	1,620 (63.4%)	464 (51.4%)	1,156 (69.8%)
Loop diuretics^g^	4,696 (60.4%)	1,450 (52.2%)	3,246 (65.0%)	1,882 (2,557)	518 (57.4%)	1,364 (82.4%)
<40 mg furosemide equivalent	15,44 (19.9%)	640 (23.0%)	904 (18.1%)	761 (29.9%)	236 (26.2%)	525 (31.7%)
40-80 mg furosemide equivalent	2,054 (26.4%)	649 (23.4%)	1,405 (28.2%)	762 (29.8%)	222 (24.6%)	530 (32.6%)
>80 mg furosemide equivalent	1,845 (23.8%)	273 (9.8%)	1,572 (31.5%)	161 (6.3%)	24 (2.7%)	137 (8.3%)
Dose not available				198 (7.7%)	36 (4.0%)	137 (8.3%)
Mineralocorticoid receptor antagonists^h^	1,638 (21.1%)	370 (13.3%)	1,268 (25.4%)	585 (22.9%)	126 (14.0%)	459 (27.7%)
Sodium glucose co-transporter 2 inhibitors^i^	303 (3.9%)	69 (2.5%)	234 (4.7%)	199 (7.8%)	53 (5.9%)	146 (8.8%)
Thiazides^j^	1,436 (18.5%)	569 (20.5%)	867 (17.4%)	475 (18.6%)	206 (22.8%)	269 (16.3%)
Estimated glomerular filtration rate, mean (SD)	—	—	—	66 (18)	69 (18)	64 (18)

Values are median (IQR), n (%), or mean (SD).

ACEi = angiotensin-converting enzyme inhibitor; ARB = angiotensin receptor blocker; ARNi = angiotensin receptor-neprilysin inhibitor; CDC SVI = Centers for Disease Control and Prevention Social Vulnerability Index; No. = number; VHA = Veterans Health Administration.

a Includes American Indian/Alaska native, Asian, Pacific Islander.

b Based on VHA rural classification system.

c The CDC Social Vulnerability Index is a measure of community risk at the census tract level ranging from 0 to 1; a score of 0.75 indicates that the neighborhood is more socially vulnerable than 75% of neighborhoods in the country.

d The proportion of patients recorded to be taking medication in each category within 6 months after the index diagnosis.

e ACEi, ARB, ARNi include captopril, moexipril, trandolapril, lisinopril, benazepril, enalapril, ramipril, fosinopril, quinapril, perindopril, losartan, valsartan, irebesartan, telmisartan, candesartan, eprosartan, olmesartan, azilsartan, and sacubitril-valsartan.

f Includes evidence-based beta-blocker for heart failure: carvedilol, bisoprolol, or metoprolol succinate.

g Includes furosemide, torsemide, bumetanide, and ethacrynic acid.

h Mineralocorticoid receptor antagonists include spironolactone and epleronone.

i Sodium glucose co-transporter 2 inhibitors include bexagliflozin, canagliflozin, dapagliflozin, empagliflozin, ertugliflozin, sotagliflozin.

j Thiazides include chlorothiazide, chlorthalidone, hydrochlorothiazide, indapamide, metolazone.

Most Medicare beneficiaries were diagnosed with ATTR-CM more than 6 months after their initial HF diagnosis (4,992; 64%). Patient characteristics are stratified by time to diagnosis using a binary cut point (>6 months) (Table 1). The median time to amyloid diagnosis was 494 days (IQR: 63, 1,340). The overall incidence of composite death or HFH was 35.8 per 100 patient-years. The incidence of death was 25.9 per 100 patient-years.

At the time of ATTR-CM diagnosis, Medicare beneficiaries with longer time to diagnosis (as compared to shorter) had higher rates of markers of disease progression such as atrial fibrillation (3,637; 72.9% vs 1,367; 49.2%), chronic kidney disease (2,744; 55.0% vs 856; 30.8%), frailty scores (1,387; 27.8% vs 448; 16.1%), pacemaker implantation (220; 4.4% vs 32;1.2%), overall rates of loop diuretics (3,246; 65.0% vs 1,450; 52.2%), and high loop diuretic dosing (1,572; 31.5% vs 273; 9.8%). Overall, 2,297 (29.6%) of Medicare beneficiaries were prescribed ATTR-CM disease-modifying therapies (DMT). 37.5% (2,287/6,100) of Medicare beneficiaries diagnosed with ATTR-CM after January 1, 2019, were prescribed DMT (Supplemental Table 4).

### VHA cohort

The median age was 81 years, with most Veterans being male (99.5%). Most Veterans identified as White (1,440 Veterans; 56.3%) or Black (764 veterans; 29.9%). The most common comorbidities at the time of amyloidosis diagnosis were hypertension (2,405 veterans; 94.1%), coronary artery disease (1,637 veterans; 64%), and chronic kidney disease (1,314 veterans; 51.4%). The most frequently prescribed cardiac medications were loop diuretics (1,882 veterans; 73.6%), beta-blockers (1,620 veterans; 63.4%), and renin-angiotensin system inhibitors (1,526 veterans; 59.7%). Baseline characteristics at the time of amyloidosis diagnosis are shown in Table 1.

Most Veterans were diagnosed with ATTR-CM more than 6 months after their initial HF diagnosis (1,655 veterans; 65%). The median time to amyloid diagnosis was 490 days (IQR: 69, 1,286) (Table 2). More than 25% of Veterans had a time to diagnosis exceeding 3 years.

**Table 2:** Association Between Time From HF Diagnosis to ATTR-CM Diagnosis and Other Secondary Outcomes

	Medicare Cohort (n = 7,770)			VHA Cohort (n = 2,557)		
	HR	95% CI	*P* Value	HR	95% CI	*P* Value
All-cause death	1.07	1.06–1.09	<0.0001	1.08	1.06–1.10	<0.0001
HFH	1.08	1.06–1.09	<0.0001	1.07	1.04–1.09	<0.0001
HFH/CV death	—	—	—	1.07	1.05–1.09	<0.0001
CV death	—	—	—	1.10	1.05–1.14	<0.0001

CV death = cardiovascular death; HF = heart failure; HFH = heart failure hospitalization; other abbreviation as in Table 1.

All models adjusted for sociodemographics: age, sex, Social Vulnerability Index, and rurality.

The overall incidence of composite death or HFH was 41.6 per 100 patient-years. The incidence of death was 25.9 per 100 patient-years.

At the time of ATTR-CM diagnosis, Veterans with a longer time to diagnosis (as compared to shorter) had higher rates of markers of disease progression such as atrial fibrillation (1,154; 69.7% vs 424; 47%), chronic kidney disease (978; 59.1% vs 336; 37.3%), frailty scores (665; 40.2% vs 216; 23.9%), pacemaker implantation (85; 5.1% vs 12; 1.3%), overall rates of loop diuretics (525; 31.7% vs 236; 26.2%), and high loop diuretic dosing (137; 8.3% vs 24; 2.7%). 938 (36.7%) of Veterans were prescribed ATTR-CM DMT. 48% (938/2,032) of Veterans diagnosed with ATTR-CM after 1/1/2019 (48%) were prescribed DMT (Supplemental Table 5).

## Association between delayed diagnosis and outcomes

### Medicare cohort

With adjustment for sociodemographics—age, sex, rurality, and social vulnerability, each year of delayed diagnosis was associated with a 7% higher risk of HFH or death with an HR of 1.07 (95% CI: 1.06-1.08). These findings were consistent after also adjusting for clinical comorbidities (HR: 1.05; 95% CI: 1.04-1.07) (Table 3). When stratified by time to diagnoses, there was a stepwise association between increase in risk with increasing diagnostic delay. In an unadjusted analysis, a delay in diagnosis of 6 months to 2 years was associated with a 44% increased risk of the primary outcome (HR: 1.44; 95% CI: 1.32-1.57) (Central Illustration). A delay in diagnosis of >2 years was associated with a 78% higher risk of the primary outcome (HR: 1.78; 95% CI: 1.66-1.92). The association between ATTR-CM time to diagnosis and mortality was similar before 2019 (HR: 1.09; 95% CI: 1.06-1.11) and between 2019 and 2022 (HR: 1.09; 95% CI: 1.07-1.10) (*P* = 0.72 for interaction).

**Table 3:** Association Between Time From Heart Failure Diagnosis (per Year) to ATTR-CM Diagnosis and Death or Heart Failure Hospitalization

	Medicare Cohort (n = 7,770)			VHA Cohort (n = 2,557)		
	HR	95% CI	*P* Value	HR	95% CI	*P* Value
Unadjusted	1.08	1.07–1.09	<0.0001	1.07	1.05–1.09	<0.0001
Adjusted for sociodemographics^a^	1.07	1.06–1.08	<0.0001	1.07	1.05–1.09	<0.0001
Adjusted for demographics and comorbidities^b^	1.05	1.04–1.07	<0.0001	1.04	1.02–1.06	<0.0001

Abbreviation as in Table 1.

a Adjusted for age, sex, Social Vulnerability Index, and rurality.

b Adjusted for age, sex, Social Vulnerability Index, rurality, chronic obstructive pulmonary disease, depression, diabetes mellitus, hypertension, liver disease, psychotic disorder, thyroid disease, and Charlson Comorbidity Index.

**Central Illustration:**
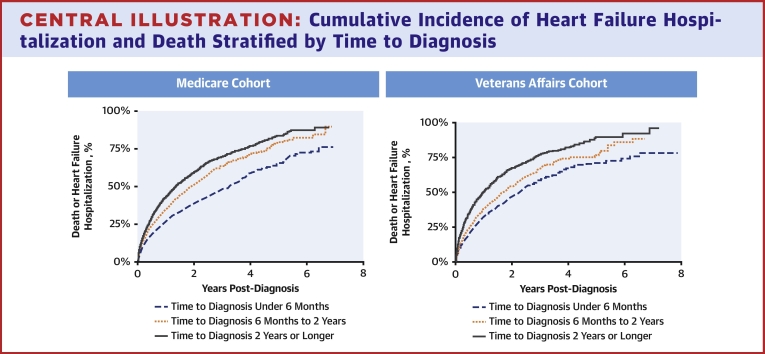
Cumulative Incidence of Heart Failure Hospitalization and Death Stratified by Time to Diagnosis

The findings were also consistent for secondary outcomes (Table 2). After adjustment for sociodemographics, each year of delayed diagnosis was associated with an 8% higher risk of HFH (HR: 1.08; 95% CI: 1.06-1.09) and a 7% higher risk of death (HR: 1.07; 95% CI: 1.06-1.09). The time from loop diuretic to ATTR-CM was also consistently associated with the risk of death. In a model only adjusted for sociodemographics, each year to diagnosis was associated with a 4% higher risk of HFH or death (HR: 1.04; 95% CI: 1.03-1.05).

### VHA cohort

In the unadjusted analysis, each year of delayed diagnosis was associated with a 7% higher risk of the primary outcome of HFH or death (HR: 1.07; 95% CI: 1.05-1.09). After adjustment for sociodemographics—age, sex, rurality, and social vulnerability, each year of delayed diagnosis was associated with a 7% higher risk of the primary outcome of HFH or death (HR: 1.07; 95% CI: 1.05-1.09). These findings were similar after adjustment for the Charlson Comorbidity Index and comorbidities (HR: 1.04; 95% CI: 1.02-1.06) (Table 4). In the unadjusted analysis, when stratified by time to diagnoses, a delay in diagnosis of 6 months to 2 years was associated with a 24% increased risk of the primary outcome (HR: 1.24; 95% CI: 1.09-1.42) (Central Illustration). A delay in diagnosis of >2 years was associated with a 68% higher risk of the primary outcome (HR: 1.68; 95% CI: 1.51-1.88). The association between ATTR-CM time to diagnosis and mortality was similar before 2019 (HR: 1.05; 95% CI: 1.02-1.09) and between 2019 and 2022 (HR: 1.08; 95% CI: 1.06-1.10; *P* = 0.25 for interaction).

**Table 4:** Association Between Time From Loop Diuretic to ATTR-CM Diagnosis and Death or Heart Failure Hospitalization

	Medicare Cohort (n = 6,175)			VHA Cohort (n = 2,030)		
	HR	95% CI	*P* Value	HR	95% CI	*P* Value
Unadjusted	1.05	1.04–1.06	<0.0001	1.02	1.01–1.03	0.001
Adjusted for sociodemographics^a^	1.04	1.03–1.05	<0.0001	1.02	1.01–1.03	0.001
Adjusted for demographics and comorbidities^b^	1.03	1.02–1.04	<0.0001	1.01	1.00–1.02	0.101

Abbreviation as in Table 1.

a Adjusted for age, sex, Social Vulnerability Index, and rurality.

b Adjusted for age, sex, Social Vulnerability Index, rurality, chronic obstructive pulmonary disease, depression, diabetes mellitus, hypertension, liver disease, psychotic disorder, thyroid disease, and Charlson Comorbidity Index.

The findings were consistent for the secondary outcomes (Table 2). Each year of delayed diagnosis was associated with a 7% increase in risk of the composite outcome of HFH and cardiovascular death (HR: 1.07; 95% CI: 1.05-1.09). With adjustment for sociodemographics, each year of delayed diagnosis was associated with a 10% increase in the risk of cardiovascular death (HR: 1.10; 95% CI: 1.05-1.14). The time from loop diuretic to ATTR-CM was also consistently associated with the risk of death. In a model only adjusted for sociodemographics, each year between loop diuretic and diagnosis was associated with a 2% increase in the risk of death (HR: 1.02; 95% CI: 1.01-1.03). The results were similar after excluding the 458 Medicare beneficiaries and 167 Veterans with ATTR-CM diagnosis prior to HF diagnosis (Supplemental Table 6). Supplemental Figure 3 displays the curvilinear association between time to diagnosis and risk of death or HFH. For both cohorts, the association increases with increasing time to diagnosis but then peaks at approximately 5 years prior to subsequent attenuation.

## Discussion

In this national study of patients with ATTR-CM, using a previously validated algorithm for identifying ATTR-CM, we identified over 10,000 individuals with ATTR-CM across Medicare and the VHA. Many of these individuals had a prolonged period from incident HF to ATTR-CM diagnosis over 1 year. Our findings support a conceptual framework in which diagnostic delay is associated with disease progression. In both cohorts, a delay in diagnosis was associated with increased HFH and mortality, with a >60% increase in risk for patients in whom diagnosis is delayed 2 years or more. The observed stepwise relationship between delay duration and outcomes and the concordant findings between the 2 cohorts further reinforces a dose-response association between delayed diagnosis and disease progression.

ATTR-CM is a fatal disease with a median survival of approximately 3.5 to 5 years.^16^, ^17^, ^18^ A recent review summarizing available data regarding the impact of delayed diagnosis on clinical outcomes did not identify any studies reporting on the association between delayed diagnosis and mortality. Nevertheless, prior studies suggest that delayed diagnosis is associated with higher NYHA class, conduction delay, and atrial fibrillation at the time of diagnosis, all measures of delayed diagnosis. However, the impact of delayed diagnosis on surrogate cardiac markers, such as N-terminal pro-brain natriuretic peptide and left ventricular ejection fraction, has been variable.^6^^,^^19^

To our knowledge, our analysis is the first to illustrate that diagnostic delay is associated with poor clinical outcomes in 2 large, real-world cohorts. We found a significant association between each year of delayed diagnosis and HFH and mortality both independently and as a composite outcome. Recent randomized trials show that targeted therapies improve survival and suggest that patients who start targeted therapies at a lower NYHA class may reap greater benefits.^20^, ^21^, ^22^ Therefore, we expected that the higher risk of mortality with delayed diagnosis would be more pronounced in the period after the Food and Drug Administration approval of targeted therapies for ATTR-CM than in patients diagnosed earlier. However, our findings were similar before and after 2019. We also found low overall rates of DMT for patients diagnosed in 2019, and after with fewer than 50% of patients receiving DMT in either the Medicare or the VHA cohort. This figure is similar to other United States-based studies and a report from Portugal^23^, ^24^, ^25^ but lower than recent reports from Germany.^26^ A plausible explanation for these low rates and null association is that our study period was too soon after the approval of targeted therapies to allow for broad therapy dissemination in the VHA and Medicare populations and to be able to appreciate the effects of these medications, which are typically observed over years. It is also possible that the delay in diagnosis and disease progression are the drivers of outcomes and the effect of the targeted therapies was not detectable in a large cohort where not all patients received targeted therapies.

### Study limitations

Our findings should be interpreted in the context of several limitations. The Medicare database only includes data for patients older than 65 years; therefore, we could not evaluate delay to diagnosis in younger non-Veterans. This is particularly pertinent for patients with variant ATTR who may present at a younger age. We did not have data on the ATTR subtype or genetic factors, which may be associated with cardiovascular outcomes.^1^^,^^27^, ^28^, ^29^ Given that this is a retrospective cohort study, there are possibly unidentified confounders that are associated both with delayed diagnosis and poorer clinical outcomes that may partially explain the association between delayed diagnosis and HFH and mortality. Furthermore, given patients had to survive from the time of HF diagnosis until ATTR-CM diagnosis, those with a longer time to diagnosis may have an inherent survival advantage compared with individuals who die prior to ATTR-CM diagnosis. This survivorship bias may lead to an underestimation of the negative association between delayed time to diagnosis and subsequent outcomes. Our study provides a modest estimate of diagnostic delay, as we use time from diagnosis of HF, rather than the time from onset of patient-reported symptoms to define delay. This cohort likely represents the tip of the iceberg regarding the association between delayed diagnosis and clinical outcomes, as we are unable to account for patients who were never diagnosed with ATTR-CM.

### Conclusions

In this nationwide study of Medicare beneficiaries and Veterans seen at the VHA with ATTR-CM, we found that a delay between the initial diagnosis of HF and subsequent identification of amyloidosis was associated with an increased risk of HFH and mortality in both cohorts independently. These findings support diagnostic delay as a clinically meaningful marker of disease progression, with longer delays reflecting more advanced disease at presentation and translating into worse outcomes.

Perspectives **COMPETENCY IN CLINICAL KNOWLEDGE:** Among patients with transthyretin cardiac amyloidosis (ATTR-CM), prolonged time from heart failure (HF) diagnosis to ATTR-CM diagnosis is associated with a substantially higher risk of subsequent HF hospitalization and mortality. These findings underscore that diagnostic delay is a clinically meaningful marker of disease severity, reinforcing the importance of early recognition of ATTR-CM in patients with HF.

**TRANSLATIONAL OUTLOOK:** Prospective studies are needed to identify patient- and health system–level barriers contributing to delayed diagnosis and to determine the impact of earlier detection in the era of emerging ATTR-directed therapies. Future studies should also evaluate whether strategies that decrease diagnostic delay, including systematic screening of high-risk HF populations, increased recognition of ATTR-CM red flags, and implementation of diagnostic algorithms, can improve timely initiation of disease-modifying therapies and translate into reductions in HF hospitalization and mortality.

## Funding support and author disclosures

This work is funded by AstraZeneca. Dr Spencer-Bonilla is supported by the 10.13039/100000968AHA supplement #24DIVSUP1258344 and 10.13039/100000050NIH/NHLBI grant 1F32HL176160-01. Dr Varshney has received consulting fees from Broadview Ventures and ECHAS. Dr Rodriguez holds equity from Carta Healthcare and HealthPals; and has received consulting fees from HealthPals, Novartis, Novo Nordisk, Esperion Therapeutics, Movano Health, Kento Health, Inclusive Health, Edwards, Arrowhead Pharmaceuticals, HeartFlow, iRhythm, Amgen, and Cleerly Health outside the submitted work. Drs Papas, Davies, Venditto, and Huang are employees and stockholders of AstraZeneca. Dr Witteles has received consulting fees (modest) from Pfizer, Alnylam, AstraZeneca, Alexion, Novo Nordisk, and Bridge Bio. Dr Alexander has received consulting fees (modest) from Arbor Biotechnologies, Attralus, Novo Nordisk, Prothena, and Pfizer. Dr Sandhu has received research funding from AHA, NIH, Amgen, Bayer Pharmaceuticals, Novo Nordisk, and Novartis Pharmaceuticals unrelated to this work; and has received consulting fees from Cleerly, Holosis Health, iRhythm, and Reprieve Cardiovascular unrelated to this work. All other authors have reported that they have no relationships relevant to the contents of this paper to disclose.
