# Supplementary material for: Delayed Diagnosis of Transthyretin Cardiac Amyloidosis Is Associated With Heart Failure Hospitalizations and Mortality
Source: JACC Adv. 2026 Jul 22;5(8):103019. doi: 10.1016/j.jacadv.2026.103019 (PMC13425842; doi:10.1016/j.jacadv.2026.103019)
Supplement: Supplemental Material [file mmc1.docx]

**Supplement Figure 1. Medicare Cohort Diagram** **of Study Inclusion and Exclusion Criteria**

Medicare enrollees with TTR CA based on structured algorithm from 2016-2022 (n=12,146)

Noncontinuous Medicare Part A, B and C enrollment in the three years prior to CA or HF diagnosis (n=1,975)

Medicare enrollees with CA and continuous enrollment in Medicare part A, B, and C (n=10,171)

Noncontinuous Medicare Part D enrollment in the 6 months prior to CA or HF diagnosis (n=2,401)

Final study cohort (n=7,770)

Abbreviations: HF: heart failure; TTR CA: Transthyretin cardiac amyloidosis.

**Supplement Figure 2. VHA Cohort Diagram of Study Inclusion and Exclusion Criteria**

No HF diagnosis (n=8,886)

Excluded based on structured TTR algorithm (n= 7,060)

Other amyloid diagnosis code (n=876)

ATTR diagnosis code or ATTR-specific therapy (n=1,681)

Final study cohort (n=2,557)

Veterans with ATTR and heart failure (n=3,866)

Veterans with first amyloid diagnosis between 2016-2022 (n=20,040)

Veterans with HF diagnosis before or within 1 year of incident amyloid diagnosis (n=10,926)

Excluded based on lack of active VA care (n=1,077)

Excluded with >10 years between incident HF and amyloid (n=232)

Abbreviations: ATTR: Transthyretin Amyloidosis; HF: Heart failure; VA: Veterans Affairs.

**Supplement Figure 3. Spline-Based Analysis of Time to Diagnosis and Death or Heart Failure Hospitalization After Excluding Those with ATTR-CM Diagnosis Before Heart Failure Diagnosis**

1. **VHA Cohort**

1. **Medicare Cohort**

**
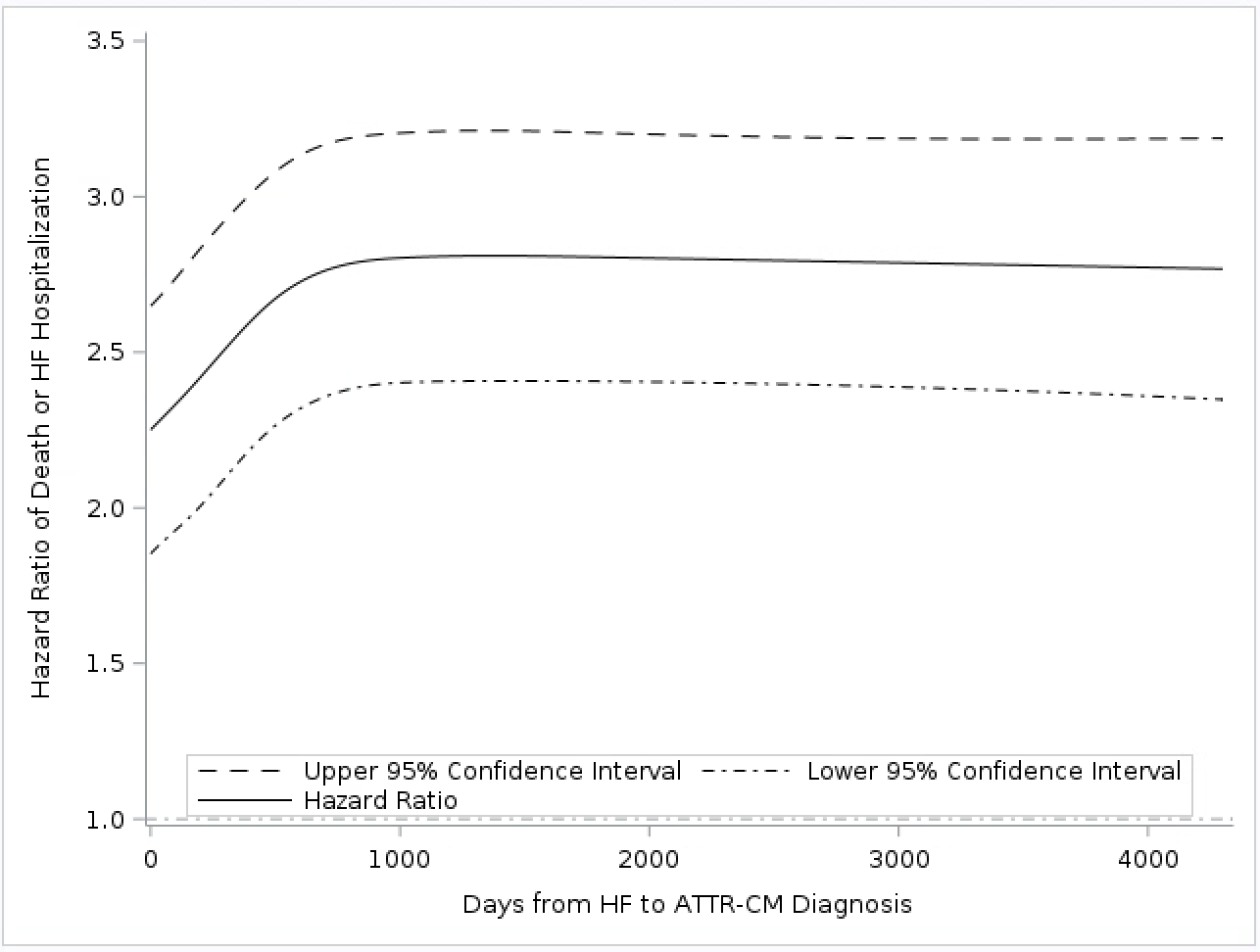
**

**Supplement Table 1. ICD Algorithm Diagnosis Codes and Medications**

|  | **Definition** |
| --- | --- |
| ATTR diagnosis | Wild-type transthyretin-related (ATTR) amyloidosis E85.82 |
| ATTR-specific therapy | Tafamidis, Diflunisal |
| No alternate etiology of amyloid | autoimmune diseases M05.X, M06.X M07.X M08.X M32.X, M45.X M46.X K50.X K51.X 714.0 555.X 556.X 710.0, 714.0 714.1 714.2 714.3X 720.0 277.31  cerebral amyloid I680  myeloma C90.0X C90.1X 203.X  end-stage renal disease N18.6 585.6 Z94.0 Z94.2 Z99.2 Z49.X V42.0 V56.X |
| No AL amyloid-specific therapy | Daratumumab, Cyclophosphamide, Carfilzomib, Thalidomide Bortezomib Pomalidomide, Melphalan, Lenolidomide |
| Any amyloid diagnosis | E85.X, 277.X |

**Supplement Table 2. ICD Codes for Medical Comorbidities**

| Alcohol use disorder | \| ICD9 \| 2652 \| \| --- \| --- \| \| ICD9 \| 2911 \| \| ICD9 \| 2912 \| \| ICD9 \| 2913 \| \| ICD9 \| 2915 \| \| ICD9 \| 2919 \| \| ICD9 \| 3575 \| \| ICD9 \| 4255 \| \| ICD9 \| 5710 \| \| ICD9 \| 5711 \| \| ICD9 \| 5712 \| \| ICD9 \| 5713 \| \| ICD9 \| 9800 \| \| ICD9 \| 9801 \| \| ICD9 \| 9802 \| \| ICD9 \| 9803 \| \| ICD9 \| 9808 \| \| ICD9 \| 9809 \| \| ICD9 \| 29181 \| \| ICD9 \| 29182 \| \| ICD9 \| 29189 \| \| ICD9 \| 30300 \| \| ICD9 \| 30301 \| \| ICD9 \| 30302 \| \| ICD9 \| 30303 \| \| ICD9 \| 30390 \| \| ICD9 \| 30391 \| \| ICD9 \| 30392 \| \| ICD9 \| 30393 \| \| ICD9 \| 30500 \| \| ICD9 \| 30501 \| | \| ICD9 \| 30502 \| \| --- \| --- \| \| ICD9 \| 30503 \| \| ICD9 \| 53530 \| \| ICD9 \| 53531 \| \| ICD9 \| V113 \| \| ICD10 \| F1011 \| \| ICD10 \| F10120 \| \| ICD10 \| F10121 \| \| ICD10 \| F10129 \| \| ICD10 \| F10130 \| \| ICD10 \| F10131 \| \| ICD10 \| F10132 \| \| ICD10 \| F10139 \| \| ICD10 \| F1014 \| \| ICD10 \| F10150 \| \| ICD10 \| F10151 \| \| ICD10 \| F10159 \| \| ICD10 \| F10180 \| \| ICD10 \| F10181 \| \| ICD10 \| F10182 \| \| ICD10 \| F10188 \| \| ICD10 \| F1019 \| \| ICD10 \| F1020 \| \| ICD10 \| F1021 \| \| ICD10 \| F10220 \| \| ICD10 \| F10221 \| \| ICD10 \| F10229 \| \| ICD10 \| F10230 \| \| ICD10 \| F10231 \| \| ICD10 \| F10232 \| \| ICD10 \| F10239 \| | \| ICD10 \| F1024 \| \| --- \| --- \| \| ICD10 \| F10250 \| \| ICD10 \| F10251 \| \| ICD10 \| F10259 \| \| ICD10 \| F1026 \| \| ICD10 \| F1027 \| \| ICD10 \| F10280 \| \| ICD10 \| F10281 \| \| ICD10 \| F10282 \| \| ICD10 \| F10288 \| \| ICD10 \| F1029 \| \| ICD10 \| F1094 \| \| ICD10 \| F10950 \| \| ICD10 \| F10951 \| \| ICD10 \| F10959 \| \| ICD10 \| F1096 \| \| ICD10 \| F1097 \| \| ICD10 \| F10980 \| \| ICD10 \| G621 \| \| ICD10 \| I426 \| \| ICD10 \| K2920 \| \| ICD10 \| K2921 \| \| ICD10 \| K7010 \| \| ICD10 \| K7011 \| \| ICD10 \| O99310 \| \| ICD10 \| O99311 \| \| ICD10 \| O99312 \| \| ICD10 \| O99313 \| \| ICD10 \| O99314 \| \| ICD10 \| O99315 \| |
| --- | --- | --- | --- | --- | --- | --- | --- | --- | --- | --- | --- | --- | --- | --- | --- | --- | --- | --- | --- | --- | --- | --- | --- | --- | --- | --- | --- | --- | --- | --- | --- | --- | --- | --- | --- | --- | --- | --- | --- | --- | --- | --- | --- | --- | --- | --- | --- | --- | --- | --- | --- | --- | --- | --- | --- | --- | --- | --- | --- | --- | --- | --- | --- | --- | --- | --- | --- | --- | --- | --- | --- | --- | --- | --- | --- | --- | --- | --- | --- | --- | --- | --- | --- | --- | --- | --- | --- | --- | --- | --- | --- | --- | --- | --- | --- | --- | --- | --- | --- | --- | --- | --- | --- | --- | --- | --- | --- | --- | --- | --- | --- | --- | --- | --- | --- | --- | --- | --- | --- | --- | --- | --- | --- | --- | --- | --- | --- | --- | --- | --- | --- | --- | --- | --- | --- | --- | --- | --- | --- | --- | --- | --- | --- | --- | --- | --- | --- | --- | --- | --- | --- | --- | --- | --- | --- | --- | --- | --- | --- | --- | --- | --- | --- | --- | --- | --- | --- | --- | --- | --- | --- | --- | --- | --- | --- | --- | --- | --- | --- | --- | --- | --- | --- | --- | --- | --- | --- |
| Aortic stenosis | \| ICD9 \| 4241 \| \| --- \| --- \| \| ICD10 \| I350 \| | \| ICD10 \| I351 \| \| --- \| --- \| \| ICD10 \| I352 \| | \| ICD10 \| I358 \| \| --- \| --- \| \| ICD10 \| I359 \| |
| Atrial fibrillation/flutter | \| ICD9 \| 42731 \| \| --- \| --- \| \| ICD9 \| 42732 \| \| ICD10 \| I48 \| \| ICD10 \| I480 \| \| ICD10 \| I481 \| | \| ICD10 \| I4811 \| \| --- \| --- \| \| ICD10 \| I4819 \| \| ICD10 \| I482 \| \| ICD10 \| I4820 \| \| ICD10 \| I4821 \| | \| ICD10 \| I483 \| \| --- \| --- \| \| ICD10 \| I484 \| \| ICD10 \| I489 \| \| ICD10 \| I4891 \| \| ICD10 \| I4892 \| |
| Carpal tunnel | \| ICD9 \| 3540 \| \| --- \| --- \| \| ICD10 \| G5600 \| | \| ICD10 \| G5601 \| \| --- \| --- \| \| ICD10 \| G5602 \| | \| ICD10 \| G5603 \| \| --- \| --- \| |
| Chronic kidney disease | \| ICD9 \| 586 \| \| --- \| --- \| \| ICD9 \| 5851 \| \| ICD9 \| 5852 \| \| ICD9 \| 5853 \| \| ICD9 \| 5854 \| \| ICD9 \| 5855 \| \| ICD9 \| 5856 \| \| ICD9 \| 5859 \| \| ICD9 \| 5880 \| \| ICD9 \| 40300 \| \| ICD9 \| 40301 \| \| ICD9 \| 40310 \| \| ICD9 \| 40311 \| \| ICD9 \| 40390 \| \| ICD9 \| 40391 \| \| ICD9 \| 40400 \| \| ICD9 \| 40401 \| \| ICD9 \| 40402 \| \| ICD9 \| 40403 \| | \| ICD9 \| 40410 \| \| --- \| --- \| \| ICD9 \| 40411 \| \| ICD9 \| 40412 \| \| ICD9 \| 40413 \| \| ICD9 \| 40490 \| \| ICD9 \| 40491 \| \| ICD9 \| 40492 \| \| ICD9 \| 40493 \| \| ICD10 \| I120 \| \| ICD10 \| I1311 \| \| ICD10 \| I132 \| \| ICD10 \| N183 \| \| ICD10 \| N1830 \| \| ICD10 \| N1831 \| \| ICD10 \| N1832 \| \| ICD10 \| N184 \| \| ICD10 \| N185 \| \| ICD10 \| N186 \| \| ICD10 \| N189 \| | \| ICD10 \| N19 \| \| --- \| --- \| \| ICD9 \| V420 \| \| ICD9 \| V4511 \| \| ICD9 \| V4512 \| \| ICD9 \| V560 \| \| ICD9 \| V561 \| \| ICD9 \| V562 \| \| ICD9 \| V5631 \| \| ICD9 \| V5632 \| \| ICD9 \| V568 \| \| ICD10 \| Z4901 \| \| ICD10 \| Z4902 \| \| ICD10 \| Z4931 \| \| ICD10 \| Z4932 \| \| ICD10 \| Z9115 \| \| ICD10 \| Z940 \| \| ICD10 \| Z992 \| |
| Chronic obstructive pulmonary disease | \| ICD9 \| 490 \| \| --- \| --- \| \| ICD9 \| 4910 \| \| ICD9 \| 4911 \| \| ICD9 \| 4912 \| \| ICD9 \| 49120 \| \| ICD9 \| 49121 \| \| ICD9 \| 49122 \| \| ICD9 \| 4918 \| \| ICD9 \| 4919 \| \| ICD9 \| 4920 \| \| ICD9 \| 4928 \| | \| ICD9 \| 494 \| \| --- \| --- \| \| ICD9 \| 4940 \| \| ICD9 \| 4941 \| \| ICD9 \| 496 \| \| ICD10 \| J40 \| \| ICD10 \| J410 \| \| ICD10 \| J411 \| \| ICD10 \| J418 \| \| ICD10 \| J42 \| \| ICD10 \| J430 \| \| ICD10 \| J431 \| | \| ICD10 \| J432 \| \| --- \| --- \| \| ICD10 \| J438 \| \| ICD10 \| J439 \| \| ICD10 \| J440 \| \| ICD10 \| J441 \| \| ICD10 \| J449 \| \| ICD10 \| J470 \| \| ICD10 \| J471 \| \| ICD10 \| J479 \| |
| Complete heart block | \| ICD9 \| I4260 \| \| --- \| --- \| | \| ICD10 \| I442 \| \| --- \| --- \| |  |
| Coronary artery disease | \| ICD9 \| 412 \| \| --- \| --- \| \| ICD9 \| 41400 \| \| ICD9 \| 41401 \| \| ICD9 \| 41402 \| \| ICD9 \| 41403 \| \| ICD9 \| 41404 \| \| ICD9 \| 41405 \| \| ICD9 \| 41406 \| \| ICD9 \| 41407 \| \| ICD9 \| 41412 \| \| ICD9 \| 4142 \| \| ICD9 \| 4143 \| \| ICD9 \| 4144 \| \| ICD9 \| 4148 \| \| ICD9 \| 4149 \| \| ICD9 \| 4292 \| \| ICD10 \| I2510 \| \| ICD10 \| I25111 \| | \| ICD10 \| I25118 \| \| --- \| --- \| \| ICD10 \| I25119 \| \| ICD10 \| I252 \| \| ICD10 \| I255 \| \| ICD10 \| I256 \| \| ICD10 \| I25701 \| \| ICD10 \| I25708 \| \| ICD10 \| I25709 \| \| ICD10 \| I25711 \| \| ICD10 \| I25718 \| \| ICD10 \| I25719 \| \| ICD10 \| I25721 \| \| ICD10 \| I25728 \| \| ICD10 \| I25729 \| \| ICD10 \| I25731 \| \| ICD10 \| I25738 \| \| ICD10 \| I25739 \| \| ICD10 \| I25751 \| | \| ICD10 \| I25758 \| \| --- \| --- \| \| ICD10 \| I25759 \| \| ICD10 \| I25761 \| \| ICD10 \| I25768 \| \| ICD10 \| I25769 \| \| ICD10 \| I25791 \| \| ICD10 \| I25798 \| \| ICD10 \| I25799 \| \| ICD10 \| I25810 \| \| ICD10 \| I25811 \| \| ICD10 \| I25812 \| \| ICD10 \| I2582 \| \| ICD10 \| I2583 \| \| ICD10 \| I2584 \| \| ICD10 \| I2589 \| \| ICD10 \| I259 \| |
| Depression | \| ICD9 \| 29620 \| \| --- \| --- \| \| ICD9 \| 29621 \| \| ICD9 \| 29622 \| \| ICD9 \| 29623 \| \| ICD9 \| 29624 \| \| ICD9 \| 29625 \| \| ICD9 \| 29626 \| \| ICD9 \| 29630 \| \| ICD9 \| 29631 \| \| ICD9 \| 29632 \| \| ICD9 \| 29633 \| \| ICD9 \| 29634 \| \| ICD9 \| 29635 \| \| ICD9 \| 29636 \| \| ICD9 \| 29650 \| \| ICD9 \| 29651 \| \| ICD9 \| 29652 \| \| ICD9 \| 29653 \| \| ICD9 \| 29655 \| | \| ICD9 \| 29656 \| \| --- \| --- \| \| ICD9 \| 3004 \| \| ICD9 \| 3090 \| \| ICD9 \| 3091 \| \| ICD9 \| 30921 \| \| ICD9 \| 30922 \| \| ICD9 \| 30923 \| \| ICD9 \| 30924 \| \| ICD9 \| 30928 \| \| ICD9 \| 30929 \| \| ICD9 \| 3093 \| \| ICD9 \| 3094 \| \| ICD9 \| 30981 \| \| ICD9 \| 30982 \| \| ICD9 \| 30983 \| \| ICD9 \| 30989 \| \| ICD9 \| 3099 \| \| ICD9 \| 311 \| \| ICD10 \| F0631 \| | \| ICD10 \| F0632 \| \| --- \| --- \| \| ICD10 \| F0634 \| \| ICD10 \| F320 \| \| ICD10 \| F321 \| \| ICD10 \| F322 \| \| ICD10 \| F323 \| \| ICD10 \| F328 \| \| ICD10 \| F3281 \| \| ICD10 \| F3289 \| \| ICD10 \| F329 \| \| ICD10 \| F32A \| \| ICD10 \| F330 \| \| ICD10 \| F331 \| \| ICD10 \| F332 \| \| ICD10 \| F333 \| \| ICD10 \| F338 \| \| ICD10 \| F339 \| \| ICD10 \| F341 \| |
| Diabetes mellitus | \| ICD9 \| 25000 \| \| --- \| --- \| \| ICD9 \| 25001 \| \| ICD9 \| 25002 \| \| ICD9 \| 25003 \| \| ICD9 \| 25010 \| \| ICD9 \| 25011 \| \| ICD9 \| 25012 \| \| ICD9 \| 25013 \| \| ICD9 \| 25020 \| \| ICD9 \| 25021 \| \| ICD9 \| 25022 \| \| ICD9 \| 25023 \| \| ICD9 \| 25030 \| \| ICD9 \| 25031 \| \| ICD9 \| 25032 \| \| ICD9 \| 25033 \| \| ICD9 \| 25040 \| \| ICD9 \| 25041 \| \| ICD9 \| 25042 \| \| ICD9 \| 25043 \| \| ICD9 \| 25050 \| \| ICD9 \| 25051 \| \| ICD9 \| 25052 \| \| ICD9 \| 25053 \| \| ICD9 \| 25060 \| \| ICD9 \| 25061 \| \| ICD9 \| 25062 \| \| ICD9 \| 25063 \| \| ICD9 \| 25070 \| \| ICD9 \| 25071 \| \| ICD9 \| 25072 \| \| ICD9 \| 25073 \| \| ICD9 \| 25080 \| \| ICD9 \| 25081 \| \| ICD9 \| 25082 \| \| ICD9 \| 25083 \| \| ICD9 \| 25090 \| \| ICD9 \| 25091 \| \| ICD9 \| 25092 \| \| ICD9 \| 25093 \| \| ICD10 \| E08 \| \| ICD10 \| E080 \| \| ICD10 \| E0800 \| \| ICD10 \| E0801 \| \| ICD10 \| E081 \| \| ICD10 \| E0810 \| \| ICD10 \| E0811 \| \| ICD10 \| E082 \| \| ICD10 \| E0821 \| \| ICD10 \| E0822 \| \| ICD10 \| E0829 \| \| ICD10 \| E083 \| \| ICD10 \| E0831 \| \| ICD10 \| E08311 \| \| ICD10 \| E08319 \| \| ICD10 \| E0832 \| \| ICD10 \| E08321 \| \| ICD10 \| E083211 \| \| ICD10 \| E083212 \| \| ICD10 \| E083213 \| \| ICD10 \| E083219 \| \| ICD10 \| E08329 \| \| ICD10 \| E083291 \| \| ICD10 \| E083292 \| \| ICD10 \| E083293 \| \| ICD10 \| E083299 \| \| ICD10 \| E0833 \| \| ICD10 \| E08331 \| \| ICD10 \| E083311 \| \| ICD10 \| E083312 \| \| ICD10 \| E083313 \| \| ICD10 \| E083319 \| \| ICD10 \| E08339 \| \| ICD10 \| E083391 \| \| ICD10 \| E083392 \| \| ICD10 \| E083393 \| \| ICD10 \| E083399 \| \| ICD10 \| E0834 \| \| ICD10 \| E08341 \| \| ICD10 \| E083411 \| \| ICD10 \| E083412 \| \| ICD10 \| E083413 \| \| ICD10 \| E083419 \| \| ICD10 \| E08349 \| \| ICD10 \| E083491 \| \| ICD10 \| E083492 \| \| ICD10 \| E083493 \| \| ICD10 \| E083499 \| \| ICD10 \| E0835 \| \| ICD10 \| E08351 \| \| ICD10 \| E083511 \| \| ICD10 \| E083512 \| \| ICD10 \| E083513 \| \| ICD10 \| E083519 \| \| ICD10 \| E08352 \| \| ICD10 \| E083521 \| \| ICD10 \| E083522 \| \| ICD10 \| E083523 \| \| ICD10 \| E083529 \| \| ICD10 \| E08353 \| \| ICD10 \| E083531 \| \| ICD10 \| E083532 \| \| ICD10 \| E083533 \| \| ICD10 \| E083539 \| \| ICD10 \| E08354 \| \| ICD10 \| E083541 \| \| ICD10 \| E083542 \| \| ICD10 \| E083543 \| \| ICD10 \| E083549 \| \| ICD10 \| E08355 \| \| ICD10 \| E083551 \| \| ICD10 \| E083552 \| \| ICD10 \| E083553 \| \| ICD10 \| E083559 \| \| ICD10 \| E08359 \| \| ICD10 \| E083591 \| \| ICD10 \| E083592 \| \| ICD10 \| E083593 \| \| ICD10 \| E083599 \| \| ICD10 \| E0836 \| \| ICD10 \| E0837 \| \| ICD10 \| E0837X1 \| \| ICD10 \| E0837X2 \| \| ICD10 \| E0837X3 \| \| ICD10 \| E0837X9 \| \| ICD10 \| E0839 \| \| ICD10 \| E084 \| \| ICD10 \| E0840 \| \| ICD10 \| E0841 \| \| ICD10 \| E0842 \| \| ICD10 \| E0843 \| \| ICD10 \| E0844 \| \| ICD10 \| E0849 \| \| ICD10 \| E085 \| \| ICD10 \| E0851 \| \| ICD10 \| E0852 \| \| ICD10 \| E0859 \| \| ICD10 \| E086 \| \| ICD10 \| E08610 \| \| ICD10 \| E08618 \| \| ICD10 \| E08620 \| \| ICD10 \| E08621 \| \| ICD10 \| E08622 \| \| ICD10 \| E08628 \| \| ICD10 \| E08630 \| \| ICD10 \| E08638 \| \| ICD10 \| E0864 \| \| ICD10 \| E08641 \| \| ICD10 \| E08649 \| \| ICD10 \| E0865 \| \| ICD10 \| E0869 \| \| ICD10 \| E088 \| \| ICD10 \| E089 \| \| ICD10 \| E09 \| \| ICD10 \| E090 \| \| ICD10 \| E0900 \| \| ICD10 \| E0901 \| \| ICD10 \| E091 \| \| ICD10 \| E0910 \| \| ICD10 \| E0911 \| \| ICD10 \| E092 \| \| ICD10 \| E0921 \| \| ICD10 \| E0922 \| \| ICD10 \| E0929 \| \| ICD10 \| E093 \| \| ICD10 \| E0931 \| \| ICD10 \| E09311 \| \| ICD10 \| E09319 \| \| ICD10 \| E0932 \| \| ICD10 \| E09321 \| \| ICD10 \| E093211 \| \| ICD10 \| E093212 \| \| ICD10 \| E093213 \| \| ICD10 \| E093219 \| \| ICD10 \| E09329 \| \| ICD10 \| E093291 \| \| ICD10 \| E093292 \| \| ICD10 \| E093293 \| \| ICD10 \| E093299 \| \| ICD10 \| E0933 \| \| ICD10 \| E09331 \| \| ICD10 \| E093311 \| \| ICD10 \| E093312 \| \| ICD10 \| E093313 \| \| ICD10 \| E093319 \| \| ICD10 \| E09339 \| \| ICD10 \| E093391 \| \| ICD10 \| E093392 \| \| ICD10 \| E093393 \| \| ICD10 \| E093399 \| \| ICD10 \| E0934 \| \| ICD10 \| E09341 \| \| ICD10 \| E093411 \| \| ICD10 \| E093412 \| \| ICD10 \| E093413 \| \| ICD10 \| E093419 \| \| ICD10 \| E09349 \| \| ICD10 \| E093491 \| \| ICD10 \| E093492 \| \| ICD10 \| E093493 \| | \| ICD10 \| E093499 \| \| \| --- \| --- \| --- \| \| ICD10 \| E0935 \| \| \| ICD10 \| E09351 \| \| \| ICD10 \| E093511 \| \| \| ICD10 \| E093512 \| \| \| ICD10 \| E093513 \| \| \| ICD10 \| E093519 \| \| \| ICD10 \| E09352 \| \| \| ICD10 \| E093521 \| \| \| ICD10 \| E093522 \| \| \| ICD10 \| E093523 \| \| \| ICD10 \| E093529 \| \| \| ICD10 \| E09353 \| \| \| ICD10 \| E093531 \| \| \| ICD10 \| E093532 \| \| \| ICD10 \| E093533 \| \| \| ICD10 \| E093539 \| \| \| ICD10 \| E09354 \| \| \| ICD10 \| E093541 \| \| \| ICD10 \| E093542 \| \| \| ICD10 \| E093543 \| \| \| ICD10 \| E093549 \| \| \| ICD10 \| E09355 \| \| \| ICD10 \| E093551 \| \| \| ICD10 \| E093552 \| \| \| ICD10 \| E093553 \| \| \| ICD10 \| E093559 \| \| \| ICD10 \| E09359 \| \| \| ICD10 \| E093591 \| \| \| ICD10 \| E093592 \| \| \| ICD10 \| E093593 \| \| \| ICD10 \| E093599 \| \| \| ICD10 \| E0936 \| \| \| ICD10 \| E0937 \| \| \| ICD10 \| E0937X1 \| \| \| ICD10 \| E0937X2 \| \| \| ICD10 \| E0937X3 \| \| \| ICD10 \| E0937X9 \| \| \| ICD10 \| E0939 \| \| \| ICD10 \| E094 \| \| \| ICD10 \| E0940 \| \| \| ICD10 \| E0941 \| \| \| ICD10 \| E0942 \| \| \| ICD10 \| E0943 \| \| \| ICD10 \| E0944 \| \| \| ICD10 \| E0949 \| \| \| ICD10 \| E095 \| \| \| ICD10 \| E0951 \| \| \| ICD10 \| E0952 \| \| \| ICD10 \| E0959 \| \| \| ICD10 \| E096 \| \| \| ICD10 \| E09610 \| \| \| ICD10 \| E09618 \| \| \| ICD10 \| E09620 \| \| \| ICD10 \| E09621 \| \| \| ICD10 \| E09622 \| \| \| ICD10 \| E09628 \| \| \| ICD10 \| E09630 \| \| \| ICD10 \| E09638 \| \| \| ICD10 \| E0964 \| \| \| ICD10 \| E09641 \| \| \| ICD10 \| E09649 \| \| \| ICD10 \| E0965 \| \| \| ICD10 \| E0969 \| \| \| ICD10 \| E098 \| \| \| ICD10 \| E099 \| \| \| ICD10 \| E1010 \| \| \| ICD10 \| E1011 \| \| \| ICD10 \| E1021 \| \| \| ICD10 \| E1022 \| \| \| ICD10 \| E1029 \| \| \| ICD10 \| E10311 \| \| \| ICD10 \| E10319 \| \| \| ICD10 \| E10321 \| \| \| ICD10 \| E103211 \| \| \| ICD10 \| E103212 \| \| \| ICD10 \| E103213 \| \| \| ICD10 \| E103219 \| \| \| ICD10 \| E10329 \| \| \| ICD10 \| E103291 \| \| \| ICD10 \| E103292 \| \| \| ICD10 \| E103293 \| \| \| ICD10 \| E103299 \| \| \| ICD10 \| E10331 \| \| \| ICD10 \| E103311 \| \| \| ICD10 \| E103312 \| \| \| ICD10 \| E103313 \| \| \| ICD10 \| E103319 \| \| \| ICD10 \| E10339 \| \| \| ICD10 \| E103391 \| \| \| ICD10 \| E103392 \| \| \| ICD10 \| E103393 \| \| \| ICD10 \| E103399 \| \| \| ICD10 \| E10341 \| \| \| ICD10 \| E103411 \| \| \| ICD10 \| E103412 \| \| \| ICD10 \| E103413 \| \| \| ICD10 \| E103419 \| \| \| ICD10 \| E10349 \| \| \| ICD10 \| E103491 \| \| \| ICD10 \| E103492 \| \| \| ICD10 \| E103493 \| \| \| ICD10 \| E103499 \| \| \| ICD10 \| E10351 \| \| \| ICD10 \| E103511 \| \| \| ICD10 \| E103512 \| \| \| ICD10 \| E103513 \| \| \| ICD10 \| E103519 \| \| \| ICD10 \| E103521 \| \| \| ICD10 \| E103522 \| \| \| ICD10 \| E103523 \| \| \| ICD10 \| E103529 \| \| \| ICD10 \| E103531 \| \| \| ICD10 \| E103532 \| \| \| ICD10 \| E103533 \| \| \| ICD10 \| E103539 \| \| \| ICD10 \| E103541 \| \| \| ICD10 \| E103542 \| \| \| ICD10 \| E103543 \| \| \| ICD10 \| E103549 \| \| \| ICD10 \| E103551 \| \| \| ICD10 \| E103552 \| \| \| ICD10 \| E103553 \| \| \| ICD10 \| E103559 \| \| \| ICD10 \| E10359 \| \| \| ICD10 \| E103591 \| \| \| ICD10 \| E103592 \| \| \| ICD10 \| E103593 \| \| \| ICD10 \| E103599 \| \| \| ICD10 \| E1036 \| \| \| ICD10 \| E1037X1 \| \| \| ICD10 \| E1037X2 \| \| \| ICD10 \| E1037X3 \| \| \| ICD10 \| E1037X9 \| \| \| ICD10 \| E1039 \| \| \| ICD10 \| E1040 \| \| \| ICD10 \| E1041 \| \| \| ICD10 \| E1042 \| \| \| ICD10 \| E1043 \| \| \| ICD10 \| E1044 \| \| \| ICD10 \| E1049 \| \| \| ICD10 \| E1051 \| \| \| ICD10 \| E1052 \| \| \| ICD10 \| E1059 \| \| \| ICD10 \| E10610 \| \| \| ICD10 \| E10618 \| \| \| ICD10 \| E10620 \| \| \| ICD10 \| E10621 \| \| \| ICD10 \| E10622 \| \| \| ICD10 \| E10628 \| \| \| ICD10 \| E10630 \| \| \| ICD10 \| E10638 \| \| \| ICD10 \| E10641 \| \| \| ICD10 \| E10649 \| \| \| ICD10 \| E1065 \| \| \| ICD10 \| E1069 \| \| \| ICD10 \| E108 \| \| \| ICD10 \| E109 \| \| \| ICD10 \| E1100 \| \| \| ICD10 \| E1101 \| \| \| ICD10 \| E1110 \| \| \| ICD10 \| E1111 \| \| \| ICD10 \| E1121 \| \| \| ICD10 \| E1122 \| \| \| ICD10 \| E1129 \| \| \| ICD10 \| E11311 \| \| \| ICD10 \| E11319 \| \| \| ICD10 \| E11321 \| \| \| ICD10 \| E113211 \| \| \| ICD10 \| E113212 \| \| \| ICD10 \| E113213 \| \| \| ICD10 \| E113219 \| \| \| ICD10 \| E11329 \| \| \| ICD10 \| E113291 \| \| \| ICD10 \| E113292 \| \| \| ICD10 \| E113293 \| \| \| ICD10 \| E113299 \| \| \| ICD10 \| E11331 \| \| \| ICD10 \| E113311 \| \| \| ICD10 \| E113312 \| \| \| ICD10 \| E113313 \| \| \| ICD10 \| E113319 \| \| \| ICD10 \| E11339 \| \| \| ICD10 \| E113391 \| \| \| ICD10 \| E113392 \| \| \| ICD10 \| E113393 \| \| \| ICD10 \| E113399 \| \| \| ICD10 \| E11341 \| \| \| ICD10 \| E113411 \| \| \| ICD10 \| E113412 \| \| \| ICD10 \| E113413 \| \| \| ICD10 \| E113419 \| \| \| ICD10 \| E11349 \| \| \| ICD10 \| E113491 \| \| \| ICD10 \| E113492 \| \| \| ICD10 \| E113493 \| \| \| ICD10 \| E113499 \| \| \| ICD10 \| E11351 \| \| \| ICD10 \| E113511 \| \| \| ICD10 \| E113512 \| | \| ICD10 \| E113513 \| \| --- \| --- \| \| ICD10 \| E113519 \| \| ICD10 \| E113521 \| \| ICD10 \| E113522 \| \| ICD10 \| E113523 \| \| ICD10 \| E113529 \| \| ICD10 \| E113531 \| \| ICD10 \| E113532 \| \| ICD10 \| E113533 \| \| ICD10 \| E113539 \| \| ICD10 \| E113541 \| \| ICD10 \| E113542 \| \| ICD10 \| E113543 \| \| ICD10 \| E113549 \| \| ICD10 \| E113551 \| \| ICD10 \| E113552 \| \| ICD10 \| E113553 \| \| ICD10 \| E113559 \| \| ICD10 \| E11359 \| \| ICD10 \| E113591 \| \| ICD10 \| E113592 \| \| ICD10 \| E113593 \| \| ICD10 \| E113599 \| \| ICD10 \| E1136 \| \| ICD10 \| E1137X1 \| \| ICD10 \| E1137X2 \| \| ICD10 \| E1137X3 \| \| ICD10 \| E1137X9 \| \| ICD10 \| E1139 \| \| ICD10 \| E1140 \| \| ICD10 \| E1141 \| \| ICD10 \| E1142 \| \| ICD10 \| E1143 \| \| ICD10 \| E1144 \| \| ICD10 \| E1149 \| \| ICD10 \| E1151 \| \| ICD10 \| E1152 \| \| ICD10 \| E1159 \| \| ICD10 \| E11610 \| \| ICD10 \| E11618 \| \| ICD10 \| E11620 \| \| ICD10 \| E11621 \| \| ICD10 \| E11622 \| \| ICD10 \| E11628 \| \| ICD10 \| E11630 \| \| ICD10 \| E11638 \| \| ICD10 \| E11641 \| \| ICD10 \| E11649 \| \| ICD10 \| E1165 \| \| ICD10 \| E1169 \| \| ICD10 \| E118 \| \| ICD10 \| E119 \| \| ICD10 \| E1300 \| \| ICD10 \| E1301 \| \| ICD10 \| E1310 \| \| ICD10 \| E1311 \| \| ICD10 \| E1321 \| \| ICD10 \| E1322 \| \| ICD10 \| E1329 \| \| ICD10 \| E13311 \| \| ICD10 \| E13319 \| \| ICD10 \| E13321 \| \| ICD10 \| E133211 \| \| ICD10 \| E133212 \| \| ICD10 \| E133213 \| \| ICD10 \| E133219 \| \| ICD10 \| E13329 \| \| ICD10 \| E133291 \| \| ICD10 \| E133292 \| \| ICD10 \| E133293 \| \| ICD10 \| E133299 \| \| ICD10 \| E13331 \| \| ICD10 \| E133311 \| \| ICD10 \| E133312 \| \| ICD10 \| E133313 \| \| ICD10 \| E133319 \| \| ICD10 \| E13339 \| \| ICD10 \| E133391 \| \| ICD10 \| E133392 \| \| ICD10 \| E133393 \| \| ICD10 \| E133399 \| \| ICD10 \| E13341 \| \| ICD10 \| E133411 \| \| ICD10 \| E133412 \| \| ICD10 \| E133413 \| \| ICD10 \| E133419 \| \| ICD10 \| E13349 \| \| ICD10 \| E133491 \| \| ICD10 \| E133492 \| \| ICD10 \| E133493 \| \| ICD10 \| E133499 \| \| ICD10 \| E13351 \| \| ICD10 \| E133511 \| \| ICD10 \| E133512 \| \| ICD10 \| E133513 \| \| ICD10 \| E133519 \| \| ICD10 \| E133521 \| \| ICD10 \| E133522 \| \| ICD10 \| E133523 \| \| ICD10 \| E133529 \| \| ICD10 \| E133531 \| \| ICD10 \| E133532 \| \| ICD10 \| E133533 \| \| ICD10 \| E133539 \| \| ICD10 \| E133541 \| \| ICD10 \| E133542 \| \| ICD10 \| E133543 \| \| ICD10 \| E133549 \| \| ICD10 \| E133551 \| \| ICD10 \| E133552 \| \| ICD10 \| E133553 \| \| ICD10 \| E133559 \| \| ICD10 \| E13359 \| \| ICD10 \| E133591 \| \| ICD10 \| E133592 \| \| ICD10 \| E133593 \| \| ICD10 \| E133599 \| \| ICD10 \| E1336 \| \| ICD10 \| E1337X1 \| \| ICD10 \| E1337X2 \| \| ICD10 \| E1337X3 \| \| ICD10 \| E1337X9 \| \| ICD10 \| E1339 \| \| ICD10 \| E1340 \| \| ICD10 \| E1341 \| \| ICD10 \| E1342 \| \| ICD10 \| E1343 \| \| ICD10 \| E1344 \| \| ICD10 \| E1349 \| \| ICD10 \| E1351 \| \| ICD10 \| E1352 \| \| ICD10 \| E1359 \| \| ICD10 \| E13610 \| \| ICD10 \| E13618 \| \| ICD10 \| E13620 \| \| ICD10 \| E13621 \| \| ICD10 \| E13622 \| \| ICD10 \| E13628 \| \| ICD10 \| E13630 \| \| ICD10 \| E13638 \| \| ICD10 \| E13641 \| \| ICD10 \| E13649 \| \| ICD10 \| E1365 \| \| ICD10 \| E1369 \| \| ICD10 \| E138 \| \| ICD10 \| E139 \| \| ICD10 \| O240 \| \| ICD10 \| O2401 \| \| ICD10 \| O24011 \| \| ICD10 \| O24012 \| \| ICD10 \| O24013 \| \| ICD10 \| O24019 \| \| ICD10 \| O2402 \| \| ICD10 \| O2403 \| \| ICD10 \| O241 \| \| ICD10 \| O2411 \| \| ICD10 \| O24111 \| \| ICD10 \| O24112 \| \| ICD10 \| O24113 \| \| ICD10 \| O24119 \| \| ICD10 \| O2412 \| \| ICD10 \| O2413 \| \| ICD10 \| O243 \| \| ICD10 \| O2431 \| \| ICD10 \| O24311 \| \| ICD10 \| O24312 \| \| ICD10 \| O24313 \| \| ICD10 \| O24319 \| \| ICD10 \| O2432 \| \| ICD10 \| O2433 \| \| ICD10 \| O2441 \| \| ICD10 \| O24410 \| \| ICD10 \| O24414 \| \| ICD10 \| O24415 \| \| ICD10 \| O24419 \| \| ICD10 \| O2442 \| \| ICD10 \| O24420 \| \| ICD10 \| O24424 \| \| ICD10 \| O24425 \| \| ICD10 \| O24429 \| \| ICD10 \| O2443 \| \| ICD10 \| O24430 \| \| ICD10 \| O24434 \| \| ICD10 \| O24435 \| \| ICD10 \| O24439 \| \| ICD10 \| O24811 \| \| ICD10 \| O24812 \| \| ICD10 \| O24813 \| \| ICD10 \| O24819 \| \| ICD10 \| O2482 \| \| ICD10 \| O2483 \| \| ICD10 \| O249 \| \| ICD10 \| O2491 \| \| ICD10 \| O24911 \| \| ICD10 \| O24912 \| \| ICD10 \| O24913 \| \| ICD10 \| O24919 \| \| ICD10 \| O2492 \| \| ICD10 \| O2493 \| |
| Drug use disorder | \| ICD9 \| 2920 \| \| --- \| --- \| \| ICD9 \| 2922 \| \| ICD9 \| 2929 \| \| ICD9 \| 29211 \| \| ICD9 \| 29212 \| \| ICD9 \| 29281 \| \| ICD9 \| 29282 \| \| ICD9 \| 29283 \| \| ICD9 \| 29284 \| \| ICD9 \| 29285 \| \| ICD9 \| 29289 \| \| ICD9 \| 30400 \| \| ICD9 \| 30401 \| \| ICD9 \| 30402 \| \| ICD9 \| 30403 \| \| ICD9 \| 30410 \| \| ICD9 \| 30411 \| \| ICD9 \| 30412 \| \| ICD9 \| 30413 \| \| ICD9 \| 30420 \| \| ICD9 \| 30421 \| \| ICD9 \| 30422 \| \| ICD9 \| 30423 \| \| ICD9 \| 30430 \| \| ICD9 \| 30431 \| \| ICD9 \| 30432 \| \| ICD9 \| 30433 \| \| ICD9 \| 30440 \| \| ICD9 \| 30441 \| \| ICD9 \| 30442 \| \| ICD9 \| 30443 \| \| ICD9 \| 30450 \| \| ICD9 \| 30451 \| \| ICD9 \| 30452 \| \| ICD9 \| 30453 \| \| ICD9 \| 30460 \| \| ICD9 \| 30461 \| \| ICD9 \| 30462 \| \| ICD9 \| 30463 \| \| ICD9 \| 30470 \| \| ICD9 \| 30471 \| \| ICD9 \| 30472 \| \| ICD9 \| 30473 \| \| ICD9 \| 30480 \| \| ICD9 \| 30481 \| \| ICD9 \| 30482 \| \| ICD9 \| 30483 \| \| ICD9 \| 30490 \| \| ICD9 \| 30491 \| \| ICD9 \| 30492 \| \| ICD9 \| 30493 \| \| ICD9 \| 30520 \| \| ICD9 \| 30521 \| \| ICD9 \| 30522 \| \| ICD9 \| 30523 \| \| ICD9 \| 30530 \| \| ICD9 \| 30531 \| \| ICD9 \| 30532 \| \| ICD9 \| 30533 \| \| ICD9 \| 30540 \| \| ICD9 \| 30541 \| \| ICD9 \| 30542 \| \| ICD9 \| 30543 \| \| ICD9 \| 30550 \| \| ICD9 \| 30551 \| \| ICD9 \| 30552 \| \| ICD9 \| 30553 \| \| ICD9 \| 30560 \| \| ICD9 \| 30561 \| \| ICD9 \| 30562 \| \| ICD9 \| 30563 \| \| ICD9 \| 30570 \| \| ICD9 \| 30571 \| \| ICD9 \| 30572 \| \| ICD9 \| 30573 \| \| ICD9 \| 30580 \| \| ICD9 \| 30581 \| \| ICD9 \| 30582 \| \| ICD9 \| 30583 \| \| ICD9 \| 30590 \| \| ICD9 \| 30591 \| \| ICD9 \| 30592 \| \| ICD9 \| 30593 \| \| ICD10 \| F1110 \| \| ICD10 \| F1111 \| \| ICD10 \| F11120 \| \| ICD10 \| F11121 \| \| ICD10 \| F11122 \| \| ICD10 \| F11129 \| \| ICD10 \| F1113 \| \| ICD10 \| F1114 \| \| ICD10 \| F11150 \| \| ICD10 \| F11151 \| \| ICD10 \| F11159 \| \| ICD10 \| F11181 \| \| ICD10 \| F11182 \| \| ICD10 \| F11188 \| \| ICD10 \| F1119 \| \| ICD10 \| F1120 \| \| ICD10 \| F1121 \| \| ICD10 \| F11220 \| \| ICD10 \| F11221 \| \| ICD10 \| F11222 \| \| ICD10 \| F11229 \| \| ICD10 \| F1123 \| \| ICD10 \| F1124 \| \| ICD10 \| F11250 \| \| ICD10 \| F11251 \| \| ICD10 \| F11259 \| \| ICD10 \| F11281 \| \| ICD10 \| F11282 \| \| ICD10 \| F11288 \| \| ICD10 \| F1129 \| \| ICD10 \| F1210 \| \| ICD10 \| F1211 \| | \| ICD10 \| F12120 \| \| --- \| --- \| \| ICD10 \| F12121 \| \| ICD10 \| F12122 \| \| ICD10 \| F12129 \| \| ICD10 \| F1213 \| \| ICD10 \| F12150 \| \| ICD10 \| F12151 \| \| ICD10 \| F12159 \| \| ICD10 \| F12180 \| \| ICD10 \| F12188 \| \| ICD10 \| F1219 \| \| ICD10 \| F1220 \| \| ICD10 \| F1221 \| \| ICD10 \| F12220 \| \| ICD10 \| F12221 \| \| ICD10 \| F12222 \| \| ICD10 \| F12229 \| \| ICD10 \| F1223 \| \| ICD10 \| F12250 \| \| ICD10 \| F12251 \| \| ICD10 \| F12259 \| \| ICD10 \| F12280 \| \| ICD10 \| F12288 \| \| ICD10 \| F1229 \| \| ICD10 \| F1310 \| \| ICD10 \| F1311 \| \| ICD10 \| F13120 \| \| ICD10 \| F13121 \| \| ICD10 \| F13129 \| \| ICD10 \| F13130 \| \| ICD10 \| F13131 \| \| ICD10 \| F13132 \| \| ICD10 \| F13139 \| \| ICD10 \| F1314 \| \| ICD10 \| F13150 \| \| ICD10 \| F13151 \| \| ICD10 \| F13159 \| \| ICD10 \| F13180 \| \| ICD10 \| F13181 \| \| ICD10 \| F13182 \| \| ICD10 \| F13188 \| \| ICD10 \| F1319 \| \| ICD10 \| F1320 \| \| ICD10 \| F1321 \| \| ICD10 \| F13220 \| \| ICD10 \| F13221 \| \| ICD10 \| F13229 \| \| ICD10 \| F13230 \| \| ICD10 \| F13231 \| \| ICD10 \| F13232 \| \| ICD10 \| F13239 \| \| ICD10 \| F1324 \| \| ICD10 \| F13250 \| \| ICD10 \| F13251 \| \| ICD10 \| F13259 \| \| ICD10 \| F1326 \| \| ICD10 \| F1327 \| \| ICD10 \| F13280 \| \| ICD10 \| F13281 \| \| ICD10 \| F13282 \| \| ICD10 \| F13288 \| \| ICD10 \| F1329 \| \| ICD10 \| F1410 \| \| ICD10 \| F1411 \| \| ICD10 \| F14120 \| \| ICD10 \| F14121 \| \| ICD10 \| F14122 \| \| ICD10 \| F14129 \| \| ICD10 \| F1413 \| \| ICD10 \| F1414 \| \| ICD10 \| F14150 \| \| ICD10 \| F14151 \| \| ICD10 \| F14159 \| \| ICD10 \| F14180 \| \| ICD10 \| F14181 \| \| ICD10 \| F14182 \| \| ICD10 \| F14188 \| \| ICD10 \| F1419 \| \| ICD10 \| F1420 \| \| ICD10 \| F1421 \| \| ICD10 \| F14220 \| \| ICD10 \| F14221 \| \| ICD10 \| F14222 \| \| ICD10 \| F14229 \| \| ICD10 \| F1423 \| \| ICD10 \| F1424 \| \| ICD10 \| F14250 \| \| ICD10 \| F14251 \| \| ICD10 \| F14259 \| \| ICD10 \| F14280 \| \| ICD10 \| F14281 \| \| ICD10 \| F14282 \| \| ICD10 \| F14288 \| \| ICD10 \| F1429 \| \| ICD10 \| F1510 \| \| ICD10 \| F1511 \| \| ICD10 \| F15120 \| \| ICD10 \| F15121 \| \| ICD10 \| F15122 \| \| ICD10 \| F15129 \| \| ICD10 \| F1513 \| \| ICD10 \| F1514 \| \| ICD10 \| F15150 \| \| ICD10 \| F15151 \| \| ICD10 \| F15159 \| \| ICD10 \| F15180 \| \| ICD10 \| F15181 \| \| ICD10 \| F15182 \| \| ICD10 \| F15188 \| \| ICD10 \| F1519 \| \| ICD10 \| F1520 \| \| ICD10 \| F1521 \| \| ICD10 \| F15220 \| \| ICD10 \| F15221 \| \| ICD10 \| F15222 \| | \| ICD10 \| F15229 \| \| --- \| --- \| \| ICD10 \| F1523 \| \| ICD10 \| F1524 \| \| ICD10 \| F15250 \| \| ICD10 \| F15251 \| \| ICD10 \| F15259 \| \| ICD10 \| F15280 \| \| ICD10 \| F15281 \| \| ICD10 \| F15282 \| \| ICD10 \| F15288 \| \| ICD10 \| F1529 \| \| ICD10 \| F1610 \| \| ICD10 \| F1611 \| \| ICD10 \| F16120 \| \| ICD10 \| F16121 \| \| ICD10 \| F16122 \| \| ICD10 \| F16129 \| \| ICD10 \| F1614 \| \| ICD10 \| F16150 \| \| ICD10 \| F16151 \| \| ICD10 \| F16159 \| \| ICD10 \| F16180 \| \| ICD10 \| F16183 \| \| ICD10 \| F16188 \| \| ICD10 \| F1619 \| \| ICD10 \| F1620 \| \| ICD10 \| F1621 \| \| ICD10 \| F16220 \| \| ICD10 \| F16221 \| \| ICD10 \| F16229 \| \| ICD10 \| F1624 \| \| ICD10 \| F16250 \| \| ICD10 \| F16251 \| \| ICD10 \| F16259 \| \| ICD10 \| F16280 \| \| ICD10 \| F16283 \| \| ICD10 \| F16288 \| \| ICD10 \| F1629 \| \| ICD10 \| F1810 \| \| ICD10 \| F1811 \| \| ICD10 \| F18120 \| \| ICD10 \| F18121 \| \| ICD10 \| F18129 \| \| ICD10 \| F1814 \| \| ICD10 \| F18150 \| \| ICD10 \| F18151 \| \| ICD10 \| F18159 \| \| ICD10 \| F1817 \| \| ICD10 \| F18180 \| \| ICD10 \| F18188 \| \| ICD10 \| F1819 \| \| ICD10 \| F1820 \| \| ICD10 \| F1821 \| \| ICD10 \| F18220 \| \| ICD10 \| F18221 \| \| ICD10 \| F18229 \| \| ICD10 \| F1824 \| \| ICD10 \| F18250 \| \| ICD10 \| F18251 \| \| ICD10 \| F18259 \| \| ICD10 \| F1827 \| \| ICD10 \| F18280 \| \| ICD10 \| F18288 \| \| ICD10 \| F1829 \| \| ICD10 \| F1910 \| \| ICD10 \| F1911 \| \| ICD10 \| F19120 \| \| ICD10 \| F19121 \| \| ICD10 \| F19122 \| \| ICD10 \| F19129 \| \| ICD10 \| F19130 \| \| ICD10 \| F19131 \| \| ICD10 \| F19132 \| \| ICD10 \| F19139 \| \| ICD10 \| F1914 \| \| ICD10 \| F19150 \| \| ICD10 \| F19151 \| \| ICD10 \| F19159 \| \| ICD10 \| F1916 \| \| ICD10 \| F1917 \| \| ICD10 \| F19180 \| \| ICD10 \| F19181 \| \| ICD10 \| F19182 \| \| ICD10 \| F19188 \| \| ICD10 \| F1919 \| \| ICD10 \| F1920 \| \| ICD10 \| F1921 \| \| ICD10 \| F19220 \| \| ICD10 \| F19221 \| \| ICD10 \| F19222 \| \| ICD10 \| F19229 \| \| ICD10 \| F19230 \| \| ICD10 \| F19231 \| \| ICD10 \| F19232 \| \| ICD10 \| F19239 \| \| ICD10 \| F1924 \| \| ICD10 \| F19250 \| \| ICD10 \| F19251 \| \| ICD10 \| F19259 \| \| ICD10 \| F1926 \| \| ICD10 \| F1927 \| \| ICD10 \| F19280 \| \| ICD10 \| F19281 \| \| ICD10 \| F19282 \| \| ICD10 \| F19288 \| \| ICD10 \| F1929 \| \| ICD10 \| O99320 \| \| ICD10 \| O99321 \| \| ICD10 \| O99322 \| \| ICD10 \| O99323 \| \| ICD10 \| O99324 \| \| ICD10 \| O99325 \| \| ICD9 \| V6542 \| |
| Hypertension | \| ICD9 \| 4010 \| \| --- \| --- \| \| ICD9 \| 4011 \| \| ICD9 \| 4019 \| \| ICD9 \| 40200 \| \| ICD9 \| 40201 \| \| ICD9 \| 40210 \| \| ICD9 \| 40211 \| \| ICD9 \| 40290 \| \| ICD9 \| 40291 \| \| ICD9 \| 40300 \| \| ICD9 \| 40301 \| \| ICD9 \| 40310 \| \| ICD9 \| 40311 \| \| ICD9 \| 40390 \| \| ICD9 \| 40391 \| \| ICD9 \| 40400 \| \| ICD9 \| 40401 \| | \| ICD9 \| 40402 \| \| --- \| --- \| \| ICD9 \| 40403 \| \| ICD9 \| 40410 \| \| ICD9 \| 40411 \| \| ICD9 \| 40412 \| \| ICD9 \| 40413 \| \| ICD9 \| 40490 \| \| ICD9 \| 40491 \| \| ICD9 \| 40492 \| \| ICD9 \| 40493 \| \| ICD9 \| 40501 \| \| ICD9 \| 40509 \| \| ICD9 \| 40511 \| \| ICD9 \| 40519 \| \| ICD9 \| 40591 \| \| ICD9 \| 40599 \| \| ICD10 \| I10 \| | \| ICD10 \| I110 \| \| --- \| --- \| \| ICD10 \| I119 \| \| ICD10 \| I120 \| \| ICD10 \| I129 \| \| ICD10 \| I130 \| \| ICD10 \| I1310 \| \| ICD10 \| I1311 \| \| ICD10 \| I132 \| \| ICD10 \| I150 \| \| ICD10 \| I151 \| \| ICD10 \| I152 \| \| ICD10 \| I158 \| \| ICD10 \| I159 \| \| ICD10 \| I160 \| \| ICD10 \| I161 \| \| ICD10 \| I169 \| |
| Liver disease | \| ICD9 \| 570 \| \| --- \| --- \| \| ICD9 \| 700 \| \| ICD9 \| 701 \| \| ICD9 \| 706 \| \| ICD9 \| 709 \| \| ICD9 \| 952 \| \| ICD9 \| 953 \| \| ICD9 \| 4560 \| \| ICD9 \| 4561 \| \| ICD9 \| 4568 \| \| ICD9 \| 5710 \| \| ICD9 \| 5711 \| \| ICD9 \| 5712 \| \| ICD9 \| 5713 \| \| ICD9 \| 5715 \| \| ICD9 \| 5716 \| \| ICD9 \| 5718 \| \| ICD9 \| 5719 \| \| ICD9 \| 5720 \| \| ICD9 \| 5721 \| \| ICD9 \| 5722 \| \| ICD9 \| 5723 \| \| ICD9 \| 5724 \| \| ICD9 \| 5728 \| \| ICD9 \| 5730 \| \| ICD9 \| 5731 \| \| ICD9 \| 5732 \| \| ICD9 \| 5733 \| \| ICD9 \| 5734 \| \| ICD9 \| 5735 \| \| ICD9 \| 5738 \| \| ICD9 \| 5739 \| \| ICD9 \| 7020 \| \| ICD9 \| 7021 \| \| ICD9 \| 7022 \| \| ICD9 \| 7023 \| \| ICD9 \| 7030 \| \| ICD9 \| 7031 \| \| ICD9 \| 7032 \| \| ICD9 \| 7033 \| \| ICD9 \| 7041 \| \| ICD9 \| 7043 \| \| ICD9 \| 7044 \| \| ICD9 \| 7049 \| \| ICD9 \| 7051 \| \| ICD9 \| 7052 \| | \| ICD9 \| 7053 \| \| --- \| --- \| \| ICD9 \| 7054 \| \| ICD9 \| 7059 \| \| ICD9 \| 7070 \| \| ICD9 \| 7071 \| \| ICD9 \| 9162 \| \| ICD9 \| 45620 \| \| ICD9 \| 45621 \| \| ICD9 \| 57140 \| \| ICD9 \| 57141 \| \| ICD9 \| 57142 \| \| ICD9 \| 57149 \| \| ICD9 \| 99749 \| \| ICD10 \| A5145 \| \| ICD10 \| A5274 \| \| ICD10 \| B180 \| \| ICD10 \| B181 \| \| ICD10 \| B182 \| \| ICD10 \| B188 \| \| ICD10 \| B189 \| \| ICD10 \| B190 \| \| ICD10 \| B1910 \| \| ICD10 \| B1911 \| \| ICD10 \| B1920 \| \| ICD10 \| B1921 \| \| ICD10 \| B199 \| \| ICD10 \| B251 \| \| ICD10 \| B581 \| \| ICD10 \| I8500 \| \| ICD10 \| I8501 \| \| ICD10 \| I8510 \| \| ICD10 \| I8511 \| \| ICD10 \| I864 \| \| ICD10 \| K700 \| \| ICD10 \| K7010 \| \| ICD10 \| K7011 \| \| ICD10 \| K702 \| \| ICD10 \| K7030 \| \| ICD10 \| K7031 \| \| ICD10 \| K7040 \| \| ICD10 \| K7041 \| \| ICD10 \| K709 \| \| ICD10 \| K713 \| \| ICD10 \| K714 \| \| ICD10 \| K7150 \| \| ICD10 \| K7151 \| | \| ICD10 \| K716 \| \| --- \| --- \| \| ICD10 \| K717 \| \| ICD10 \| K718 \| \| ICD10 \| K7210 \| \| ICD10 \| K7211 \| \| ICD10 \| K7290 \| \| ICD10 \| K7291 \| \| ICD10 \| K730 \| \| ICD10 \| K731 \| \| ICD10 \| K732 \| \| ICD10 \| K738 \| \| ICD10 \| K739 \| \| ICD10 \| K740 \| \| ICD10 \| K7400 \| \| ICD10 \| K7401 \| \| ICD10 \| K7402 \| \| ICD10 \| K741 \| \| ICD10 \| K742 \| \| ICD10 \| K743 \| \| ICD10 \| K744 \| \| ICD10 \| K745 \| \| ICD10 \| K7460 \| \| ICD10 \| K7469 \| \| ICD10 \| K751 \| \| ICD10 \| K752 \| \| ICD10 \| K753 \| \| ICD10 \| K754 \| \| ICD10 \| K7581 \| \| ICD10 \| K7589 \| \| ICD10 \| K759 \| \| ICD10 \| K760 \| \| ICD10 \| K761 \| \| ICD10 \| K762 \| \| ICD10 \| K763 \| \| ICD10 \| K764 \| \| ICD10 \| K765 \| \| ICD10 \| K766 \| \| ICD10 \| K767 \| \| ICD10 \| K7681 \| \| ICD10 \| K7682 \| \| ICD10 \| K7689 \| \| ICD10 \| K769 \| \| ICD10 \| K77 \| \| ICD10 \| K9182 \| \| ICD9 \| V427 \| \| ICD10 \| Z944 \| |
| Peripheral neuropathy | \| ICD9 \| 42732 \| \| --- \| --- \| | \| ICD10 \| G9009 \| \| --- \| --- \| |  |
| Psychotic Disorders/Schizophrenia | \| ICD9 \| 29381 \| \| --- \| --- \| \| ICD9 \| 29382 \| \| ICD9 \| 29383 \| \| ICD9 \| 29384 \| \| ICD9 \| 29389 \| \| ICD9 \| 29500 \| \| ICD9 \| 29501 \| \| ICD9 \| 29502 \| \| ICD9 \| 29503 \| \| ICD9 \| 29504 \| \| ICD9 \| 29505 \| \| ICD9 \| 29510 \| \| ICD9 \| 29511 \| \| ICD9 \| 29512 \| \| ICD9 \| 29513 \| \| ICD9 \| 29514 \| \| ICD9 \| 29515 \| \| ICD9 \| 29520 \| \| ICD9 \| 29521 \| \| ICD9 \| 29522 \| \| ICD9 \| 29523 \| \| ICD9 \| 29524 \| \| ICD9 \| 29525 \| \| ICD9 \| 29530 \| \| ICD9 \| 29531 \| \| ICD9 \| 29532 \| \| ICD9 \| 29533 \| \| ICD9 \| 29534 \| \| ICD9 \| 29535 \| \| ICD9 \| 29540 \| \| ICD9 \| 29541 \| \| ICD9 \| 29542 \| \| ICD9 \| 29543 \| \| ICD9 \| 29544 \| \| ICD9 \| 29545 \| \| ICD9 \| 29550 \| \| ICD9 \| 29551 \| \| ICD9 \| 29552 \| \| ICD9 \| 29553 \| \| ICD9 \| 29554 \| \| ICD9 \| 29555 \| \| ICD9 \| 29560 \| \| ICD9 \| 29561 \| \| ICD9 \| 29562 \| \| ICD9 \| 29563 \| \| ICD9 \| 29564 \| \| ICD9 \| 29565 \| \| ICD9 \| 29570 \| \| ICD9 \| 29571 \| \| ICD9 \| 29572 \| \| ICD9 \| 29573 \| \| ICD9 \| 29574 \| \| ICD9 \| 29575 \| \| ICD9 \| 29580 \| \| ICD9 \| 29581 \| \| ICD9 \| 29582 \| \| ICD9 \| 29583 \| \| ICD9 \| 29584 \| \| ICD9 \| 29585 \| \| ICD9 \| 29590 \| \| ICD9 \| 29591 \| \| ICD9 \| 29592 \| \| ICD9 \| 29593 \| \| ICD9 \| 29594 \| \| ICD9 \| 29595 \| \| ICD9 \| 29604 \| \| ICD9 \| 29614 \| \| ICD9 \| 29644 \| \| ICD9 \| 29654 \| \| ICD9 \| 2970 \| \| ICD9 \| 2971 \| \| ICD9 \| 2972 \| \| ICD9 \| 2973 \| \| ICD9 \| 2978 \| \| ICD9 \| 2979 \| \| ICD9 \| 2980 \| | \| ICD9 \| 2981 \| \| --- \| --- \| \| ICD9 \| 2982 \| \| ICD9 \| 2983 \| \| ICD9 \| 2984 \| \| ICD9 \| 2988 \| \| ICD9 \| 2989 \| \| ICD10 \| F060 \| \| ICD10 \| F061 \| \| ICD10 \| F062 \| \| ICD10 \| F0630 \| \| ICD10 \| F0633 \| \| ICD10 \| F11150 \| \| ICD10 \| F11151 \| \| ICD10 \| F11159 \| \| ICD10 \| F11250 \| \| ICD10 \| F11251 \| \| ICD10 \| F11259 \| \| ICD10 \| F11950 \| \| ICD10 \| F11951 \| \| ICD10 \| F11959 \| \| ICD10 \| F12150 \| \| ICD10 \| F12151 \| \| ICD10 \| F12159 \| \| ICD10 \| F12250 \| \| ICD10 \| F12251 \| \| ICD10 \| F12259 \| \| ICD10 \| F12950 \| \| ICD10 \| F12951 \| \| ICD10 \| F12959 \| \| ICD10 \| F13150 \| \| ICD10 \| F13151 \| \| ICD10 \| F13159 \| \| ICD10 \| F13250 \| \| ICD10 \| F13251 \| \| ICD10 \| F13259 \| \| ICD10 \| F13950 \| \| ICD10 \| F13951 \| \| ICD10 \| F13959 \| \| ICD10 \| F14150 \| \| ICD10 \| F14151 \| \| ICD10 \| F14159 \| \| ICD10 \| F14250 \| \| ICD10 \| F14251 \| \| ICD10 \| F14259 \| \| ICD10 \| F14950 \| \| ICD10 \| F14951 \| \| ICD10 \| F14959 \| \| ICD10 \| F15150 \| \| ICD10 \| F15151 \| \| ICD10 \| F15159 \| \| ICD10 \| F15250 \| \| ICD10 \| F15251 \| \| ICD10 \| F15259 \| \| ICD10 \| F15950 \| \| ICD10 \| F15951 \| \| ICD10 \| F15959 \| \| ICD10 \| F16150 \| \| ICD10 \| F16151 \| \| ICD10 \| F16159 \| \| ICD10 \| F16250 \| \| ICD10 \| F16251 \| \| ICD10 \| F16259 \| \| ICD10 \| F16950 \| \| ICD10 \| F16951 \| \| ICD10 \| F16959 \| \| ICD10 \| F18150 \| \| ICD10 \| F18151 \| \| ICD10 \| F18159 \| \| ICD10 \| F18250 \| \| ICD10 \| F18251 \| \| ICD10 \| F18259 \| \| ICD10 \| F18950 \| \| ICD10 \| F18951 \| \| ICD10 \| F18959 \| \| ICD10 \| F19150 \| \| ICD10 \| F19151 \| | \| ICD10 \| F19159 \| \| --- \| --- \| \| ICD10 \| F19250 \| \| ICD10 \| F19251 \| \| ICD10 \| F19259 \| \| ICD10 \| F19950 \| \| ICD10 \| F19951 \| \| ICD10 \| F19959 \| \| ICD10 \| F200 \| \| ICD10 \| F201 \| \| ICD10 \| F202 \| \| ICD10 \| F203 \| \| ICD10 \| F205 \| \| ICD10 \| F2081 \| \| ICD10 \| F2089 \| \| ICD10 \| F209 \| \| ICD10 \| F21 \| \| ICD10 \| F22 \| \| ICD10 \| F23 \| \| ICD10 \| F24 \| \| ICD10 \| F250 \| \| ICD10 \| F251 \| \| ICD10 \| F258 \| \| ICD10 \| F259 \| \| ICD10 \| F28 \| \| ICD10 \| F29 \| \| ICD10 \| F3010 \| \| ICD10 \| F3011 \| \| ICD10 \| F3012 \| \| ICD10 \| F3013 \| \| ICD10 \| F302 \| \| ICD10 \| F303 \| \| ICD10 \| F304 \| \| ICD10 \| F308 \| \| ICD10 \| F309 \| \| ICD10 \| F310 \| \| ICD10 \| F3110 \| \| ICD10 \| F3111 \| \| ICD10 \| F3112 \| \| ICD10 \| F3113 \| \| ICD10 \| F312 \| \| ICD10 \| F3130 \| \| ICD10 \| F3131 \| \| ICD10 \| F3132 \| \| ICD10 \| F314 \| \| ICD10 \| F315 \| \| ICD10 \| F3160 \| \| ICD10 \| F3161 \| \| ICD10 \| F3162 \| \| ICD10 \| F3163 \| \| ICD10 \| F3164 \| \| ICD10 \| F3170 \| \| ICD10 \| F3171 \| \| ICD10 \| F3172 \| \| ICD10 \| F3173 \| \| ICD10 \| F3174 \| \| ICD10 \| F3175 \| \| ICD10 \| F3176 \| \| ICD10 \| F3177 \| \| ICD10 \| F3178 \| \| ICD10 \| F3181 \| \| ICD10 \| F3189 \| \| ICD10 \| F319 \| \| ICD10 \| F324 \| \| ICD10 \| F325 \| \| ICD10 \| F3340 \| \| ICD10 \| F3341 \| \| ICD10 \| F3342 \| \| ICD10 \| F340 \| \| ICD10 \| F348 \| \| ICD10 \| F3481 \| \| ICD10 \| F3489 \| \| ICD10 \| F349 \| \| ICD10 \| F39 \| \| ICD10 \| F4489 \| \| ICD10 \| F843 \| |
| Spinal stenosis | \| ICD9 \| 8408 \| \| --- \| --- \| \| ICD9 \| 72400 \| \| ICD9 \| 72402 \| \| ICD10 \| M4800 \| \| ICD10 \| M4801 \| | \| ICD10 \| M4802 \| \| --- \| --- \| \| ICD10 \| M4803 \| \| ICD10 \| M4803 \| \| ICD10 \| M4804 \| \| ICD10 \| M4805 \| | \| ICD10 \| M4806 \| \| --- \| --- \| \| ICD10 \| M48061 \| \| ICD10 \| M4807 \| \| ICD10 \| M4808 \| |
| Thyroid disease | \| ICD9 \| 2409 \| \| --- \| --- \| \| ICD9 \| 243 \| \| ICD9 \| 2440 \| \| ICD9 \| 2441 \| \| ICD9 \| 2442 \| \| ICD9 \| 2443 \| \| ICD9 \| 2448 \| \| ICD9 \| 2449 \| \| ICD9 \| 2461 \| \| ICD9 \| 2468 \| \| ICD10 \| E000 \| \| ICD10 \| E001 \| \| ICD10 \| E002 \| \| ICD10 \| E009 \| \| ICD10 \| E010 \| \| ICD10 \| E011 \| \| ICD10 \| E012 \| \| ICD10 \| E018 \| \| ICD10 \| E02 \| \| ICD10 \| E030 \| \| ICD10 \| E031 \| \| ICD10 \| E032 \| \| ICD10 \| E033 \| \| ICD10 \| E034 \| \| ICD10 \| E035 \| \| ICD10 \| E038 \| \| ICD10 \| E039 \| \| ICD10 \| E890 \| | \| ICD9 \| 2400 \| \| --- \| --- \| \| ICD9 \| 2409 \| \| ICD9 \| 2410 \| \| ICD9 \| 2411 \| \| ICD9 \| 2419 \| \| ICD9 \| 24200 \| \| ICD9 \| 24201 \| \| ICD9 \| 24210 \| \| ICD9 \| 24211 \| \| ICD9 \| 24220 \| \| ICD9 \| 24221 \| \| ICD9 \| 24230 \| \| ICD9 \| 24231 \| \| ICD9 \| 24240 \| \| ICD9 \| 24241 \| \| ICD9 \| 24280 \| \| ICD9 \| 24281 \| \| ICD9 \| 24290 \| \| ICD9 \| 24291 \| \| ICD9 \| 2450 \| \| ICD9 \| 2451 \| \| ICD9 \| 2452 \| \| ICD9 \| 2453 \| \| ICD9 \| 2454 \| \| ICD9 \| 2458 \| \| ICD9 \| 2459 \| \| ICD9 \| 64812 \| \| ICD9 \| 64814 \| | \| ICD10 \| E040 \| \| --- \| --- \| \| ICD10 \| E041 \| \| ICD10 \| E042 \| \| ICD10 \| E048 \| \| ICD10 \| E049 \| \| ICD10 \| E0500 \| \| ICD10 \| E0501 \| \| ICD10 \| E0510 \| \| ICD10 \| E0511 \| \| ICD10 \| E0520 \| \| ICD10 \| E0521 \| \| ICD10 \| E0530 \| \| ICD10 \| E0531 \| \| ICD10 \| E0540 \| \| ICD10 \| E0541 \| \| ICD10 \| E0580 \| \| ICD10 \| E0581 \| \| ICD10 \| E0590 \| \| ICD10 \| E0591 \| \| ICD10 \| E060 \| \| ICD10 \| E061 \| \| ICD10 \| E062 \| \| ICD10 \| E063 \| \| ICD10 \| E064 \| \| ICD10 \| E065 \| \| ICD10 \| E069 \| \| ICD10 \| O905 \| |
| Valve disease | \| ICD9 \| 3940 \| \| --- \| --- \| \| ICD9 \| 3941 \| \| ICD9 \| 3942 \| \| ICD9 \| 3949 \| \| ICD9 \| 3950 \| \| ICD9 \| 3951 \| \| ICD9 \| 3952 \| \| ICD9 \| 3959 \| \| ICD9 \| 3960 \| \| ICD9 \| 3961 \| \| ICD9 \| 3962 \| \| ICD9 \| 3963 \| \| ICD9 \| 3968 \| \| ICD9 \| 3969 \| \| ICD9 \| 3970 \| \| ICD9 \| 3971 \| \| ICD9 \| 3979 \| \| ICD9 \| 4240 \| \| ICD9 \| 4241 \| \| ICD9 \| 4242 \| \| ICD9 \| 4243 \| \| ICD9 \| 7463 \| \| ICD9 \| 7464 \| \| ICD9 \| 7465 \| \| ICD9 \| 7466 \| \| ICD9 \| 9320 \| \| ICD9 \| 9321 \| \| ICD9 \| 9322 \| \| ICD9 \| 9323 \| \| ICD9 \| 9324 \| \| ICD9 \| 42490 \| \| ICD9 \| 42491 \| \| ICD9 \| 42499 \| \| ICD10 \| A1884 \| \| ICD10 \| A3282 \| \| ICD10 \| A3951 \| \| ICD10 \| A5203 \| \| ICD10 \| B3321 \| \| ICD10 \| B376 \| \| ICD10 \| I011 \| \| ICD10 \| I018 \| \| ICD10 \| I019 \| \| ICD10 \| I020 \| \| ICD10 \| I050 \| \| ICD10 \| I051 \| \| ICD10 \| I052 \| \| ICD10 \| I058 \| | \| ICD10 \| I059 \| \| --- \| --- \| \| ICD10 \| I060 \| \| ICD10 \| I061 \| \| ICD10 \| I062 \| \| ICD10 \| I068 \| \| ICD10 \| I069 \| \| ICD10 \| I070 \| \| ICD10 \| I071 \| \| ICD10 \| I072 \| \| ICD10 \| I078 \| \| ICD10 \| I079 \| \| ICD10 \| I080 \| \| ICD10 \| I081 \| \| ICD10 \| I082 \| \| ICD10 \| I083 \| \| ICD10 \| I088 \| \| ICD10 \| I089 \| \| ICD10 \| I091 \| \| ICD10 \| I0989 \| \| ICD10 \| I330 \| \| ICD10 \| I339 \| \| ICD10 \| I340 \| \| ICD10 \| I341 \| \| ICD10 \| I342 \| \| ICD10 \| I348 \| \| ICD10 \| I3481 \| \| ICD10 \| I3489 \| \| ICD10 \| I349 \| \| ICD10 \| I350 \| \| ICD10 \| I351 \| \| ICD10 \| I352 \| \| ICD10 \| I358 \| \| ICD10 \| I359 \| \| ICD10 \| I360 \| \| ICD10 \| I361 \| \| ICD10 \| I362 \| \| ICD10 \| I368 \| \| ICD10 \| I369 \| \| ICD10 \| I370 \| \| ICD10 \| I371 \| \| ICD10 \| I372 \| \| ICD10 \| I378 \| \| ICD10 \| I379 \| \| ICD10 \| I38 \| \| ICD10 \| I39 \| \| ICD10 \| M3211 \| \| ICD10 \| Q220 \| | \| ICD10 \| Q221 \| \| --- \| --- \| \| ICD10 \| Q222 \| \| ICD10 \| Q223 \| \| ICD10 \| Q224 \| \| ICD10 \| Q225 \| \| ICD10 \| Q226 \| \| ICD10 \| Q228 \| \| ICD10 \| Q229 \| \| ICD10 \| Q230 \| \| ICD10 \| Q231 \| \| ICD10 \| Q232 \| \| ICD10 \| Q233 \| \| ICD10 \| Q234 \| \| ICD10 \| Q238 \| \| ICD10 \| Q239 \| \| ICD10 \| T8201XA \| \| ICD10 \| T8201XD \| \| ICD10 \| T8201XS \| \| ICD10 \| T8202XA \| \| ICD10 \| T8202XD \| \| ICD10 \| T8202XS \| \| ICD10 \| T8203XA \| \| ICD10 \| T8203XD \| \| ICD10 \| T8203XS \| \| ICD10 \| T8209XA \| \| ICD10 \| T8209XD \| \| ICD10 \| T8209XS \| \| ICD10 \| T82221A \| \| ICD10 \| T82221D \| \| ICD10 \| T82221S \| \| ICD10 \| T82222A \| \| ICD10 \| T82222D \| \| ICD10 \| T82222S \| \| ICD10 \| T82223A \| \| ICD10 \| T82223D \| \| ICD10 \| T82223S \| \| ICD10 \| T82228A \| \| ICD10 \| T82228D \| \| ICD10 \| T82228S \| \| ICD10 \| T826XXA \| \| ICD10 \| T826XXD \| \| ICD10 \| T826XXS \| \| ICD9 \| V422 \| \| ICD9 \| V433 \| \| ICD10 \| Z952 \| \| ICD10 \| Z953 \| \| ICD10 \| Z954 \| |

**Supplement Table 3. CPT Codes for Medical Procedures**

| **Cardiac procedures** | **CPT Code** |
| --- | --- |
| Aortic valve replacement | \| 33361 \| \| --- \| \| 33362 \| \| 33363 \| \| 33364 \| \| 33365 \| \| 33366 \| \| 33367 \| \| 33368 \| \| 33405 \| \| 33406 \| \| 33410 \| \| 33411 \| \| 33412 \| |
| Atrial ablation | \| 93653 \| \| --- \| \| 93656 \| \| 93657 \| |
| Cardioversion | 92961 |
| Pacemaker or defibrillator implantation | \| 33214 \| \| --- \| \| 33216 \| \| 33217 \| \| 33221 \| \| 33224 \| \| 33225 \| \| 33227 \| \| 33228 \| \| 33229 \| \| 33230 \| \| 33240 \| \| 33249 \| \| 33369 \| |

**Supplement Table 4. Disease Modifying Therapy Prescriptions in the Medicare Population**

| **Overall** | | | | **Amyloid Date Before 1/1/19** | | | **Amyloid Date After 1/1/19** | | |  |
| --- | --- | --- | --- | --- | --- | --- | --- | --- | --- | --- |
|  |  |  |  |  |  |  |  |  |  |  |
| **Characteristics** | **Total Medicare Cohort (N=7,770)** | **Shorter Time to Diagnosis (N=2,778)** | **Longer Time to Diagnosis (N=4,992)** | **Total Medicare Cohort (N=1670)** | **Shorter Time to Diagnosis (N=663)** | **Longer Time to Diagnosis (N=1007)** | **Total Medicare Cohort (N=6100)** | **Shorter Time to Diagnosis (N=2115)** | **Longer Time to Diagnosis (N=3985)** |  |
| **TTR Amyloid Therapy (tafamidis, patisiran, or inotersen)** | 2297 (29.6%) | 854 (30.7%) | 1418 (28.9%) | ---^*^ | ---^*^ | ---^*^ | ---^*^ | ---^*^ | ---^*^ |  |
|  |  |  |  |  |  |  |  |  |  |  |
| **TTR stabilizer (tafamidis)** | 2258 (29.1%) | 840 (30.2%) | 1418 (28.4%) | ---^*^ | ---^*^ | ---^*^ | ---^*^ | ---^*^ | ---^*^ |  |
|  |  |  |  |  |  |  |  |  |  |  |
| **TTR Silencer (patisiran, inotersen)** | 54 (0.69%) | 18 (0.65%) | 36 (0.72%) | ---^*^ | ---^*^ | ---^*^ | ---^*^ | ---^*^ | ---^*^ |  |
|  |  |  |  |  |  |  |  |  |  |  |

* Unable to display given Medicare small cell size restrictions (<10) related to low rates of amyloid therapies prior to 1/1/2019.

**Supplement Table 5. Disease Modifying Therapy Prescriptions in the Veterans Health Administration**

| Overall | | | | **Amyloid Date Before 1/1/19** | | | **Amyloid Date After 1/1/19** | | |  |
| --- | --- | --- | --- | --- | --- | --- | --- | --- | --- | --- |
|  |  |  |  |  |  |  |  |  |  |  |
| **Characteristics** | **Total VHA Cohort (N=2,557)** | **Shorter Time to Diagnosis (N=902)** | **Longer Time to Diagnosis (N=1,655)** | **Total (n=525)** | **Shorter Time to Diagnosis (N=218)** | **Longer Time to Diagnosis (N=317)** | **Total (n=2,032)** | **Short Time to Diagnosis (n=684)** | **Longer Time to Diagnosis (n=1,348)** |  |
| TTR Amyloid Therapy (tafamidis, patisiran, or inotersen) | 938 (36.7%) | 331 (36.7%) | 607 (36.7%) | 0 (0%) | 0 (0%) | 0 (0%) | 938 (46.2%) | 331 (48.4%) | 607 (45.0%) |  |
|  |  |  |  |  |  |  |  |  |  |  |
| TTR stabilizer (tafamidis) | ---^†^ | ---^†^ | ---^†^ | 0 (0%) | 0 (0%) | 0 (0%) | ---^†^ | ---^†^ | ---^†^ |  |
|  |  |  |  |  |  |  |  |  |  |  |
| TTR Silencer (patisiran, inotersen) | ---^†^ | ---^†^ | ---^†^ | 0 (0%) | 0 (0%) | 0 (0%) | ---^†^ | ---^†^ | ---^†^ |  |
|  |  |  |  |  |  |  |  |  |  |  |

* Unable to display given VHA small cell size restrictions (<10) related to low rates of use of TTR silencer therapy. Rates of TTR stabilizer can not be displayed as subsequent calculation of cell sizes <10 would be feasible.

**Supplement Table 6. Association Between Time from Heart Failure Diagnosis (Per Year) to ATTR-CM Diagnosis and Death or Heart Failure Hospitalization After Excluding Those with ATTR-CM Diagnosis Before Heart Failure Diagnosis**

|  | **Medicare Cohort (n=7,312)** | | | **VHA Cohort (n=2,390)** | | |
| --- | --- | --- | --- | --- | --- | --- |
|  | **HR** | **95% CI** | **p-value** | **HR** | **95% CI** | **p-value** |
| **Unadjusted** | 1.07 | 1.06-1.08 | <0.001 | 1.06 | 1.04-1.08 | <0.001 |
| **Adjusted for sociodemographics*** | 1.06 | 1.05-1.07 | <0.001 | 1.06 | 1.04-1.08 | <0.001 |
| **Adjusted for Demographics and Comorbidities**** | 1.05 | 1.04-1.06 | <0.001 | 1.03 | 1.01-1.06 | 0.001 |

*Adjusted for age, sex, social vulnerability index, and rurality

**Adjusted for age, sex, social vulnerability index, rurality, chronic obstructive pulmonary disease, depression, diabetes mellitus, hypertension, liver disease, psychotic disorder, thyroid disease, and Charlson comorbidity index
